# Bacterial Diversity of Marine Biofilm Communities in Terra Nova Bay (Antarctica) by Culture‐Dependent and ‐Independent Approaches

**DOI:** 10.1111/1462-2920.70045

**Published:** 2025-02-02

**Authors:** Bisaccia Melissa, Binda Elisa, Caruso Gabriella, Azzaro Maurizio, Dell' Acqua Ombretta, Di Cesare Andrea, Ester Maria Eckert, Marinelli Flavia

**Affiliations:** ^1^ Department of Biotechnology and Life Sciences (DBSV) University of Insubria Varese Italy; ^2^ National Research Council Institute of Polar Sciences (ISP) Messina Italy; ^3^ Department of Sciences of the Earth, Environment and Life (DISTAV) University of Genoa Genoa Italy; ^4^ National Research Council Water Research Institute (IRSA) Verbania Italy

**Keywords:** Antarctic Ocean, anthropic impact, enrichment, enzyme bioprospecting, extreme environment, metabarcoding, microbial cultivation

## Abstract

Applying both culture‐independent and ‐dependent approaches, bacterial diversity of marine biofilm communities colonising polyvinyl chloride panels submerged in Terra Nova Bay (Ross Sea, Antarctica) was investigated. Panels were deployed in two sites subjected to a different degree of anthropogenic impact (Road Bay [RB] impacted site and Punta Stocchino [PTS] control site). Biofilm samples were collected after 3 or 12 months to evaluate both short‐ and long‐term microbial colonisation. Taxonomic composition of the microbial community was studied by 16S rRNA gene amplicon sequencing. *Proteobacteria* was the predominant phylum, followed by *Bacteroidetes*, *Actinobacteria*, *Verrucomicrobia* and *Firmicutes*. Impacted RB biofilms were found to contain a relevant fraction of potentially pathogenic bacterial genera, accounting for 27.49% of the whole community. A total of 86 psychrotolerant bacterial strains were isolated from the biofilm samples using culture‐dependent techniques designed to enrich in *Actinobacteria*. These strains were assigned to three different phyla: *Actinobacteria* (54.65%), *Firmicutes* (32.56%) and *Proteobacteria* (12.79%). 2.73% of genera identified by metabarcoding were recovered also through cultivation, while 11 additional genera were uniquely yielded by cultivation. Functional screening of the isolates revealed their hydrolytic and oxidative enzyme activity patterns, giving new insights into the metabolic and biotechnological potential of microbial biofilm communities in Terra Nova Bay seawater.

## Graphical abstract

We characterised the biodiversity of marine microbial biofilms in Terra Nova Bay, investigating their change during 1‐year colonisation of plastic panels and in response to anthropogenic influence. Both culture‐dependent and ‐independent approaches were applied to draw an integrated picture and get insights into microbial biotechnological potential through enzyme bioprospecting.

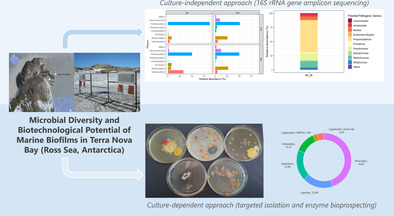

## 1 Introduction

The Antarctic continent is known as the coldest, driest, windiest and most inaccessible continent on the Earth. It is almost completely ice‐covered and isolated from other continents by the Southern Ocean and the Antarctic Circumpolar Current (Núñez‐Montero and Barrientos 2018). The Southern Ocean represents ~9.6% of the world's oceans (35 million km^2^), with an average depth of 4000 m. About 21 million km^2^ of water are ice‐covered during the winter season, but only 7 million of these persist through summer. Antarctic surface seawater temperatures annually range between −1.86°C and +0.3°C (Cavicchioli 2015; Lauritano et al. 2020). In such an extreme environment, marine microorganisms have managed to adapt and thrive, facing several challenges, including periodical limitation of nutrients and dissolved oxygen, low temperatures, freeze–thaw cycles as well as variable solar radiation (Margesin and Collins 2019). Antarctic microorganisms display unique physiological features to survive in such harsh constantly shifting conditions, including the formation of complex biofilm structures (Yin et al. 2019; Caroppo et al. 2022; Caruso et al. 2023). Biofilms are organised aggregates of microorganisms, living attached to an abiotic or biotic surface and encapsulated in a self‐produced matrix of extracellular polymeric substances (i.e., polysaccharides, proteins, lipids and nucleic acids) (Caroppo et al. 2022; Caruso et al. 2023). Biofilm lifestyle is an endless cycle, comprising the following phases: attachment, colonisation, development, maturation and active dispersal. Biofilms allow for an easier exchange of nutrients, molecules and information among the included microorganisms; their three‐dimensional structure has also been shown to act as a natural barrier and protective layer against UV radiation, extreme temperature and pH, high salinity, high pressure and nutrients paucity, proving to be a particularly advantageous life strategy to colonise extreme habitats such as Antarctica (Yin et al. 2019; Caroppo et al. 2022; Caruso et al. 2023). Despite microbial diversity of the Southern Ocean waters being previously described as comparable to that of tropical and temperate oceans (Cavicchioli 2015), Antarctic marine biofilms, which are considered hot spots of microbial diversity, are still poorly investigated. An additional reason to study these microbial communities is that their members might possess peculiar biochemical pathways and produce novel molecules, which could open the way to sustainable industrial and biotechnological applications (Rizzo and Lo Giudice 2020). For example, enzymes produced by cold‐ and halo‐tolerant marine bacteria generally show a high specific activity and remarkable stability in a wide range of variable operating conditions and solvents. This makes them a valuable alternative to labile mesophilic biocatalysts and to hazardous chemical catalysts in the food and beverage, detergent, textile, leather, pulp and paper, biofuel, cosmetic and pharmaceutical fields, as well as in bioremediation (Espina et al. 2022; Dumorné et al. 2017; Kuddus et al. 2024; Ramasamy et al. 2023; de Lourdes Moreno et al. 2013). Marine polar microorganisms were previously reported to produce novel bioactive specialised metabolites as well, and their biosurfactants and osmolytes have also attracted much interest (Núñez‐Montero and Barrientos 2018; Rizzo and Lo Giudice 2020; Poli et al. 2017).

Most of the first available reports about Antarctic marine microbial diversity and its bioprospecting potential have been based on culture‐dependent isolation, although it is widely recognised that this approach is limited by the small fraction (0.1%–1%) of microorganisms present in an environmental sample which are actually culturable (Bowman et al. 1997; Schultz et al. 2023). With the advent of culture‐independent methods and the progresses recorded in molecular microbiology, approaches such as 16S rRNA gene amplicon sequencing or shotgun sequencing have been increasingly applied to the analysis of environmental samples, giving a deeper insight into microbial communities' composition. By metabarcoding and metagenomics, in the Southern Ocean, *Gammaproteobacteria*, *Bacteroidetes* and *Alphaproteobacteria* were found to be the predominant classes of microorganisms, followed in lower percentages by *Actinobacteria*, *Epsilonproteobacteria* and *Firmicutes* (Doytchinov and Dimov 2022; Verde et al. 2016; Pikuta, Hoover, and Tang 2007; Signori et al. 2014; Papale et al. 2024). However, some commonly present culturable microbial species were reported to be frequently missed by metabarcoding approaches, which may suffer from methodological bias and sample composition interferences (Donachie, Foster, and Brown 2007; Gutleben et al. 2018). In addition, culture‐dependent isolation of microbial strains is an essential step for their functional screening and for evaluating their biotechnological potential. In fact, identification, retrieval and heterologous expression of target gene sequences directly from environmental DNA, bypassing the culturing procedures, still suffer from high costs and low efficiency, especially from extreme environments (Cowan et al. 2015; Sysoev et al. 2021). Considering the latest advances in culturing techniques (Lewis et al. 2021), a more comprehensive picture of the actual microbial diversity of the Antarctic marine regions may therefore be achieved by combining and comparing both approaches side‐by‐side.

**FIGURE 1:**
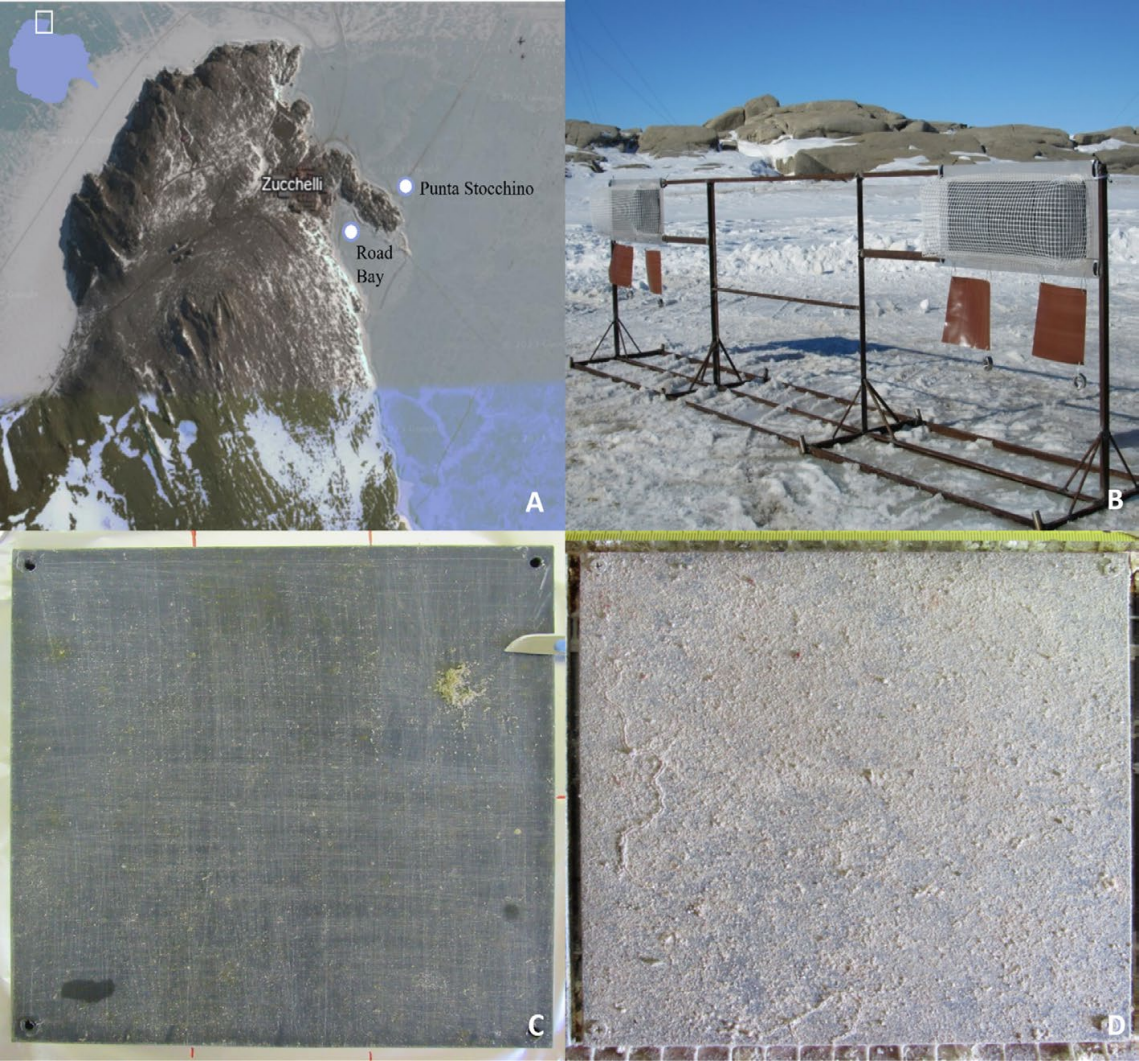
(A) Satellite image of the sampling sites in Terra Nova Bay (Ross Sea, Antarctica). Modified from Google Earth (2023, Google. U.S. Geological Survey. Data SIO, NOAA, U.S. Navy, NGA, GEBCO. PCG/NASA: NASA Landsat/Copernicus. https://www.google.it/intl/it/earth/). Road Bay (RB) site is affected by the wastewater of the Italian Mario Zucchelli (MZ) research station, while Punta Stocchino (PTS) is considered a pristine control site. (B) Stainless steel structures mounting plastic panels in polyvinyl chloride (18 cm^2^), which were submerged at −5 m in RB and PTS. PVC panels showing microbial colonisation recovered from RB after 3 months (C) or 12 months (D) of immersion.

Herein, in the frame of a project funded by the Italian National Antarctic Research Program (PNRA) on the microbial colonisation of benthic Antarctic environments (Caroppo et al. 2022; Caruso et al. 2023, 2022; Papale et al. 2024), we investigated the microbial communities' composition of marine biofilms colonising polyvinyl chloride (PVC) panels, which had been submerged in the area of Terra Nova Bay (Ross Sea, Antarctica) during the 33rd and 34th Italian Antarctic expeditions. The biofilms under investigation in this study came from panels collocated into two sites, respectively, within and just outside of Road Bay (RB), a small coastal bay close to the Italian Mario Zucchelli (MZ) research station (Figure 1). To evaluate whether anthropogenic pressure could affect the local microbial diversity, one of the two sampling sites was located close to the coast, where it was exposed to the influence of the treated wastewater effluents from the MZ station (sampling site named RB), whereas the other one, acting as the control site (sampling site named Punta Stocchino, PTS) was outside the bay, far from any possible routine human activity (Caroppo et al. 2022; Caruso et al. 2022; Bruni, Maugeri, and Monticelli 1997). Microbial community composition was assessed through 16S rRNA gene amplicon sequencing, as well as by culturing selected microbial isolates. Cultivation conditions were specifically designed to enrich for *Actinobacteria* (in particular for filamentous actinobacteria, as for instance *Streptomyces* genus), which represent a widely renowned source of bioactive macromolecules and enzymes (Rizzo and Lo Giudice 2020; Poli et al. 2017; Hui et al. 2021; Bruno et al. 2019). Filamentous actinobacteria have been thus far rarely reported from the Antarctic marine environment (Doytchinov and Dimov 2022). The obtained Antarctic bacterial isolates were therefore screened for the production of hydrolytic or oxidative enzymes involved in the degradation of proteins, lipids, carbohydrates, and lignin. Thus, this study contributes to gaining a first insight into the bioprospecting of the marine microbial diversity of the Ross Sea and its mosaic of different extreme habitats.

## 2 Experimental Procedures

### 2.1 Sample Collection and Physicochemical Analyses

During the PNRA campaign in November 2017, at the beginning of the Antarctic summer season, artificial structures in stainless steel mounting plastic panels (18 cm^2^) in PVC were deployed, through holes in the pack‐ice, at −5 m at two different sites of Terra Nova Bay: RB and PTS (Figure 1A). Before immersion, the plastic panels were cleaned with ethanol and covered with a nylon net (1 cm mesh) to protect them from large predators, which may interfere with microbial colonisation (Figure 1B). Biofilm samples growing on the panels were collected after either 3 months (3M, short‐term colonisation—January 2018) or 12 months (12M, long‐term colonisation—November 2018) of immersion (Figure 1C,D). Contextually to the deployment and subsequent samplings, the main physicochemical parameters of the surrounding water were recorded at each site using a SeaBird 9–11 multiparametric probe (SeaBird Instruments Inc., Washington, DC, USA), as previously reported by (Caroppo et al. 2022). Coordinates of the sampling sites as well as corresponding temperature, pH, salinity, and dissolved oxygen values are reported in Table 1. Water samples for nutrient analyses were collected in Niskin bottles (10 L‐volume, SCUBLA S.r.l., Remanzacco, Udine, Italy), filtered using GF/F glass‐fibre filters and stored at −20°C. Analytical determinations were performed in triplicate with a Varian Mod. Cary 50 spectrophotometer (VARIAN Inc., Palo Alto, CA, USA). The Strickland & Parsons method was applied to determine nitrite, nitrate and orthophosphate content (Strickland and Parsons 1972), while for ammonia the Aminot & Chaussepied method was followed (Aminot and Chaussepied 1983). At the MZ station laboratory, microbial biofilms were scraped from PVC panels using a sterile scalpel and avoiding contamination. They were stored at −20°C for the shipment to Italy, where they were conserved at −20°C.

**TABLE 1:** Coordinates and main water physiochemical parameters measured in Road Bay (RB) and Punta Stocchino (PTS) immediately after deployment (time 0), and after 3 or 12 months from panel immersion; nd, not determined (Caroppo et al. 2022).

Sampling site	Coordinates	Site characteristics	Depth (m)	Months	Temperature (°C)	pH	Salinity (psu)	Dissolved oxygen (mL/L)
RB	Lat 74°41.473′ S, long 164°07.125′ E	Impacted site (wastewater effluents)	−5	0	−1.83	8.02	35.19	8.0
				3	0.90	8.11	34.79	7.9
				12	−1.73	nd	35.24	nd
PTS	Lat 74°41.651′ S, Long 164°07.303′ E	Control site	−5	0	−1.82	7.93	35.22	7.2
				3	0.91	8.10	34.84	8.4

### 2.2 DNA Extraction and Sequencing

DNA from fourteen biofilm samples was extracted for 16S rRNA gene amplicon sequencing: three replicates for RB_3M (RB1GA, RB2GA, RB2GA2), four replicates for RB_12M (RB1NA, RB1NB, RB2NA, RB2NB), three replicates for PTS_3M (PTS1G, PTS1GA, PTS2G) and four replicates for PTS_12M (PTS1NB, PTS1NB1, PTS2NA, PTS2NB). Each biofilm sample was processed independently for DNA extraction using PowerSoil DNA isolation kit (Qiagen, Hilden, Germany), according to the manufacturer instructions. Negative controls were included throughout the DNA extraction procedure to assess potential contamination. DNA isolated from each sample was quantified with Qubit Fluorometer (Invitrogen, Thermo Fisher Scientific, Waltham, Massachusetts, USA), using the double‐stranded DNA HS assay kit (Invitrogen). No measurable DNA was detected in the negative controls with Qubit Fluorometer, so they were not further analysed and contamination during DNA extraction was excluded. The isolated DNA was then submitted to IGA Technology Services Srl (Udine, Italy) for 16S rRNA gene amplicon sequencing by Illumina MiSeq plaform. Universal bacterial primers S‐D‐Bact‐0341‐b‐S‐17 (forward primer, 5′‐CCTACGGGNGGCWGCAG‐3′) and S‐D‐Bact‐0785‐a‐A‐21 (reverse primer, 5′‐GACTACHVGGGTATCTAATCC‐3′) were used (Herlemann et al. 2011). All sequences were submitted to the Sequence Read Archive (SRA) of the National Center for Biotechnology Information (NCBI) under the BioProject accession number PRJNA1126177.

### 2.3 Bioinformatics and Statistical Analysis

Raw FASTQ files of 16S rRNA gene sequences were processed using USEARCH software for the clustering of operational taxonomic units (OTUs) in zero‐radius OTUs (ZOTUs) (Edgar 2010). ZOTUs were calculated using the UNOISE algorithm for Amplicon Sequence Variants, which attempts to identify all correct biological sequences in the reads (high‐quality requirements and more than eight reads in the dataset) and cluster the other sequences around them, resulting in presumed 100% sequence identity (Edgar 2016a, 2016b). Taxonomic assignments of ZOTUs were obtained by using the SILVA database (release 138.1) (Quast et al. 2013). Sequences were quality‐checked, excluding reads attributed to eukaryotes (i.e., chloroplast), reads attributed to *Archaea* (i.e., *Euryarchaeota*) and unassigned reads. They were then rarefied to the minimum number of reads per sample prior to calculation of alpha‐ and beta‐diversity statistics and further taxonomic analyses. For alpha diversity, richness of the samples and a general linear model with Poisson regression were used to evaluate and detect significant differences among the samples. The output was given as an ANOVA (analysis of variance) table from the Anova(glm) function in the car package (Sathicq et al. 2022). For beta diversity, Bray–Curtis dissimilarity index was used for principal coordinates analysis (PCoA) and the permutational multivariate analysis of variance (PERMANOVA) was applied to investigate relationships between bacterial community composition and environmental parameters (i.e., site and month). To compare the variance in microbial community with the variance in the water physiochemical parameters and nutrient content, Gower's distance for the water physiochemical parameters and nutrient content per sample was calculated using the vegan package (Oksanen et al. 2020). The samples' clustering was plotted with hclust; average linkage and PERMANOVA analysis were conducted to evaluate the effect of sampling site and month on the distances using the vegan package (Oksanen et al. 2020). The distance matrix was then correlated with the beta diversity of the community using a Mantel test. To evaluate differences between *Actinobacteria* abundance at the different sampling months, a linear model with log transformation of the read numbers was applied. The output was given as an ANOVA table transformed with the car package (Sathicq et al. 2022). Potential pathobiome analysis, investigating the presence of potential human pathogenic bacteria in the microbial biofilm communities, was performed (Fox and Weisberg 2019). Briefly, the potential pathobiome was obtained by subsetting the ZOTU table to only those ZOTUs identified up to the genus level and whose genera were found on the human pathogen list provided by the Hartmann Science Center (https://www.hartmann‐science‐center.com/en/hygiene‐knowledge/pathogens‐a‐z), mapping their presence or absence across the different types of biofilm samples. All taxonomic and statistical analyses and the resulting graphs were performed using the packages tidyverse (Wickham et al. 2019), ggplot2 (Wickham 2016), RColorBrewer (Neuwirth 2014), GUniFrac (Chen 2021), car (Sathicq et al. 2022), vegan (Oksanen et al. 2020), reshape2 (Wickham 2007) and lme4 (Bates et al. 2015) in the R studio platform (R, version 4.1.0).

### 2.4 Isolation, Purification and Preservation of Biofilm Strains

To isolate heterotrophic marine bacteria, 2.4 g of frozen biofilm samples were defrosted at 5°C and resuspended in sterile physiological solution (0.9% w/v NaCl) at a concentration of 2 mL per gram of biofilm. Half of the resuspended samples were subjected to a heat treatment of 5 min at 55°C to favour the enrichment of spore‐forming microorganisms. Samples were plated either directly or serially diluted (10^−3^, 10^−6^ and 10^−9^) on sixteen different selective solid culture media, most of them previously reported to enrich for filamentous actinobacteria (Hsu and Lockwood 1975; Marcone et al. 2017), whose composition is reported in Table S1. In all media, pH was adjusted to 7.0 before sterilisation. After sterilisation, all media were amended with nalidixic acid (25 mg/L) (Merck KGaA, Darmstadt, Germany) and cycloheximide (50 mg/L) (Merck KGaA) as selective agents to inhibit fast‐growing Gram‐negative bacteria and fungi, respectively. Isolation plates were incubated at three different temperatures: 4°C, 20°C, and 28°C for 1 month. Colonies grown on the plates were morphologically dereplicated, that is, only one isolate per plate was selected among those showing similar colony morphology, considering colour, size, shape and consistency. Sporulation was then observed under the stereomicroscope (Bel Photonics). Selected microorganisms were picked and subcultured thrice at 20°C on Marine Agar (Conda Laboratories, Madrid, Spain) to isolate axenic cultures. Purified Antarctic marine biofilm strains were observed under the microscope (Zeiss Primo Star, 40× magnification) to distinguish unicellular cocci or rods from filamentous microbes, multicellular structures, spores, etc. For Gram staining, the microorganisms were grown on Marine Agar at 20°C for 96 h and the procedure was performed according to the manufacturer's instruction with a Gram Staining Kit (Merck KGaA).

Working cell banks (WCBs) for each strain were prepared from liquid cultures grown in Tryptic Soy Broth (TSB) (Conda Laboratories) added with 2% w/v artificial sea salt (Haquoss, Aquarialand, Turin, Italy) at 20°C and 200 rpm for 96 h. WCBs were stored at −80°C adding glycerol at a final concentration of 10% v/v.

### 2.5 Biofilm Strains Genomic DNA Extraction and 16S rRNA Gene Sequencing Identification

For 16S rRNA gene sequencing, Antarctic marine biofilm strains were cultivated in 100 mL Erlenmeyer flasks containing 20 mL of TSB added with 2% w/v artificial sea salt, incubated at 20°C and 200 rpm for up to 96 h. Cells were harvested by centrifugation at 3000 g for 15 min at 4°C and genomic DNA (gDNA) was extracted from pellets according to the Kirby mix‐based protocol, as reported in (Kieser et al. 2000). gDNA quality concentration was checked at Nanodrop One/One^C^ UV–Vis (Thermo Fisher Scientific). gDNA was stored at −20°C. gDNA was sent to Macrogen (Seoul, Korea), which performed 16S rRNA gene amplification (27F/1492R universal primers), product purification and sequencing on 3730xl DNA Analyser (785F/907R universal primers). 16S rRNA gene sequences from the Antarctic marine biofilm strains were deposited at GenBank (accession numbers PQ062283‐PQ062334). A BLAST (basic local alignment search tool) search via the NCBI website was conducted with default parameters to identify the closest phylogenetically related microorganisms to the marine biofilm isolates.

### 2.6 Screening of Biofilm Strains for Hydrolytic/Oxidative Enzyme Activities

Antarctic marine biofilm isolates were screened for the detection of hydrolytic and/or oxidative enzyme activities, namely, proteolytic, lipolytic, amylolytic, chitinolytic, cellulolytic and ligninolytic activities. The screening for enzyme activities was conducted on agar plates using colorimetric and/or degradation assays, following validated protocols reported in previous works (Hsu and Lockwood 1975; Berini et al. 2016; Lal and Cheeptham 2012; Ariffin et al. 2006; Casciello et al. 2017). Solid medium composition and the type of assay used to detect each enzyme activity are reported in Table 2. Plates were incubated at 20°C for up to 3 weeks and routinely inspected. Uninoculated plates were prepared, incubated at 20°C and monitored in parallel, as negative controls.

**TABLE 2:** Enzyme assays on agar plates for the detection of hydrolytic and oxidative activities.

Enzyme activity	Assay type	Media composition (g/L)	Positive result	Reference
Proteolytic	Degradation	10 casein, 10 powdered skim milk, 15 agar, 20 artificial sea salt	Clear halo formation	Berini et al. (2016)
Lipolytic	Degradation/colorimetric	10 glyceryl‐tributyrate, 10 Arabic gum, 15 agar, 20 artificial sea salt, 1 Phenol Red	Clear halo formation and pH‐sensitive plate colour shift from red to yellow	Berini et al. (2016)
Amylolytic	Degradation	3 meat extract, 10 soluble starch, 15 agar, 20 artificial sea salt	Clear halo formation, visualised after Gram Iodine solution flooding (20 min)	Lal and Cheeptham (2012)
Chitinolytic	Degradation	4 colloidal chitin (Wickham 2016), 0.7 K_2_HPO_4_, 0.5 MgSO_4_·7 H_2_O, 0.3 KH_2_PO_4_, 0.01 FeSO_4_, 0.001 ZnSO_4_, 0.001 MnCl_2_, 20 agar, 20 artificial sea salt	Clear halo formation	Hsu and Lockwood (1975)
Cellulolytic	Degradation	5 carboxy‐methylcellulose, 1 NaNO_3_, 1 K_2_HPO_4_, 1 KCl, 1 glucose, 0.5 MgSO_4_·7 H_2_O, 0.5 yeast extract, 15 agar, 20 artificial sea salt	Clear halo formation, visualised after Congo Red (1 g/L) flooding (30 min) and destaining (30 min, 1 M NaCl, 2% v/v acetic acid)	Ariffin et al. (2006)
Ligninolytic (ABTS)	Colorimetric	20 mannitol, 2 KNO_3_, 2 MgSO_4_·7 H_2_O, 2 Na_2_HPO_4_, 15 agar, 20 artificial sea salt, 5 mM 2,2′‐Azinobis‐(3‐ethylbenzthiazolin‐6‐sulfonic acid) (ABTS)	Plate colour shift to green	Casciello et al. (2017)
Ligninolytic (Azure B)	Degradation	20 mannitol, 2 KNO_3_, 2 MgSO_4_·7 H_2_O, 2 Na_2_HPO_4_, 15 agar, 20 artificial sea salt, 25 mg/L Azure B	Clear halo formation	Casciello et al. (2017)

*Note:* Type of assay, medium composition (in g/L), and the expected positive screening results are reported. Medium pH was adjusted to 7.0. All the media components, unless otherwise stated, were obtained from Merck KGaA (artificial sea salt was from Haquoss, Aquarialand).

## 3 Results

### 3.1 Alpha‐ and Beta‐Diversity Analyses of the Antarctic Marine Microbial Biofilm Communities

Marine microbial biofilm samples (*n* = 14) were collected from PVC panels, submerged in RB and PTS in November (Figure 1A,B). Summer in Antarctica starts in October and ends in March, with winter lasting from March to October. Biofilm samples were collected after either 3 months (3M, in January, in the middle of the Antarctic summer season) or 12 months (12M, in November, after the winter period, at the beginning of the following summer season) (Figure 1C,D). Coherently with the different sampling seasons, water temperatures were over zero in January samples and below zero in November samples, with salinity also slightly lower in January during the full summertime, likely because of the higher temperatures and ice melting (Table 1). The pH was in general slightly alkaline (more in January than in November). Maximal ammonia concentration was detected in PTS in January (1.66 μM), while nitrite content peaked in RB in January (0.09 μM). Nitrate (26.05 μM in RB and 25.77 μM in PTS) and ortophosphate (1.39 μM in RB and 1.68 μM in PTS) concentrations were found higher in November at both sampling sites. Further details about the environmental parameters are reported in a previous publication (Caroppo et al. 2022).

**FIGURE 2:**
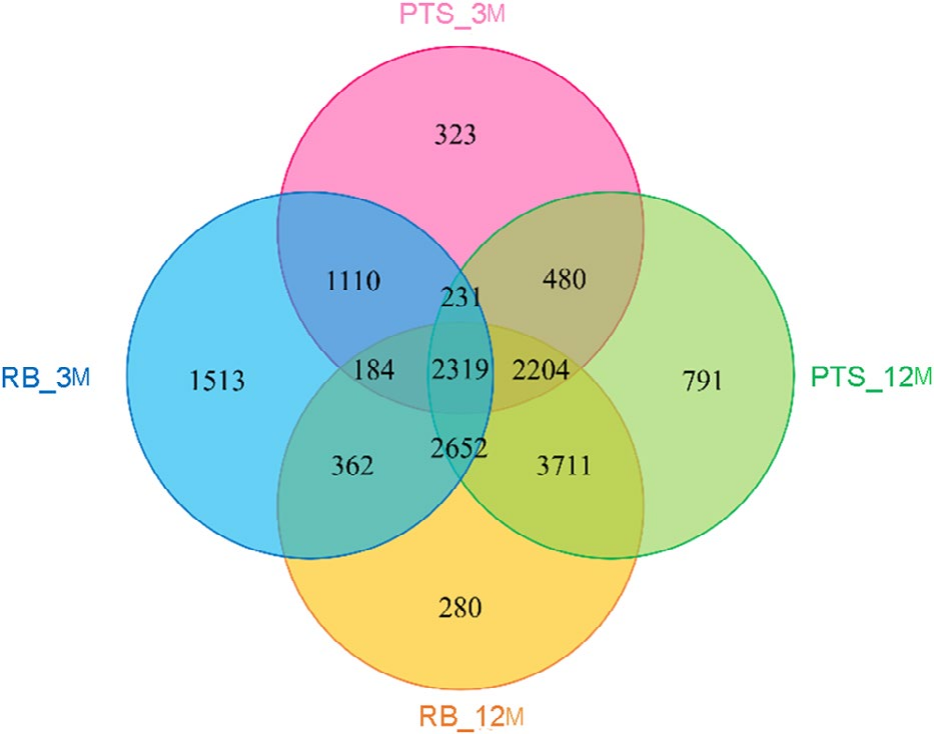
Venn diagram showing the zero‐radius operational taxonomic units (ZOTUs) shared among samples from different colonisation times (3 months, 3M, and 12 months, 12M) and sampling sites (RB and PTS). Replicate samples from each condition were considered together.

From the analysis by 16S rRNA gene amplicon sequencing of the biofilm samples from the four different conditions (i.e., RB_3M, RB_12M, PTS_3M and PTS_12M), a total of 1,836,092 reads were generated, ranging from 179,739 (PTS2NA) to 82,263 (RB1NA) reads per sample. They were rarefied to the minimum number of reads per sample. Analysis of the sequences obtained from prokaryotic amplicons resulted in 16,160 ZOTUs (8371 in RB_3M, 11,712 in RB_12M, 6851 in PTS_3M and 12,388 in PTS_12M samples). Of these, 2319 ZOTUs resulted shared among all the four conditions, while 1513, 280, 323 and 791 ZOTUs were found to be specific to RB_3M, RB_12M, PTS_3M and PTS_12M conditions, respectively (Figure 2).

**FIGURE 3:**
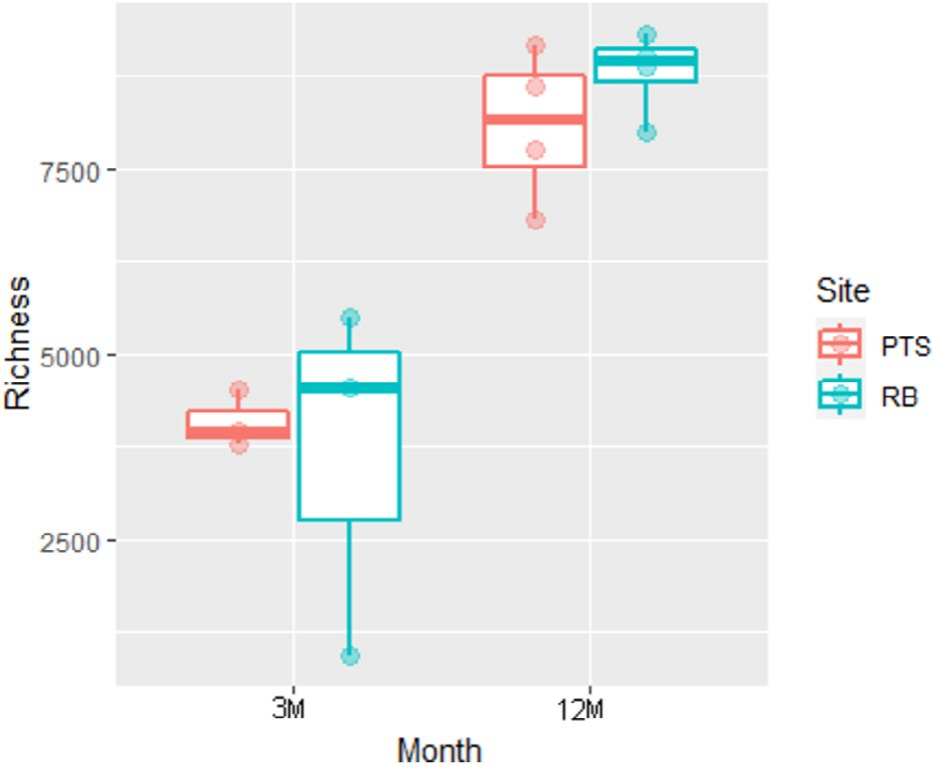
Alpha‐diversity analysis on the Antarctic marine microbial biofilm communities collected in two different sites (RB or PTS) and at different colonisation times (3 or 12M). Boxplot distribution of richness of the analysed biofilm samples expressed as number of different ZOTUs detected per sample as factor of site (RB or PTS) and colonisation time (month, 3 or 12M). Single richness values of each sample are independently shown as single points.

**FIGURE 4:**
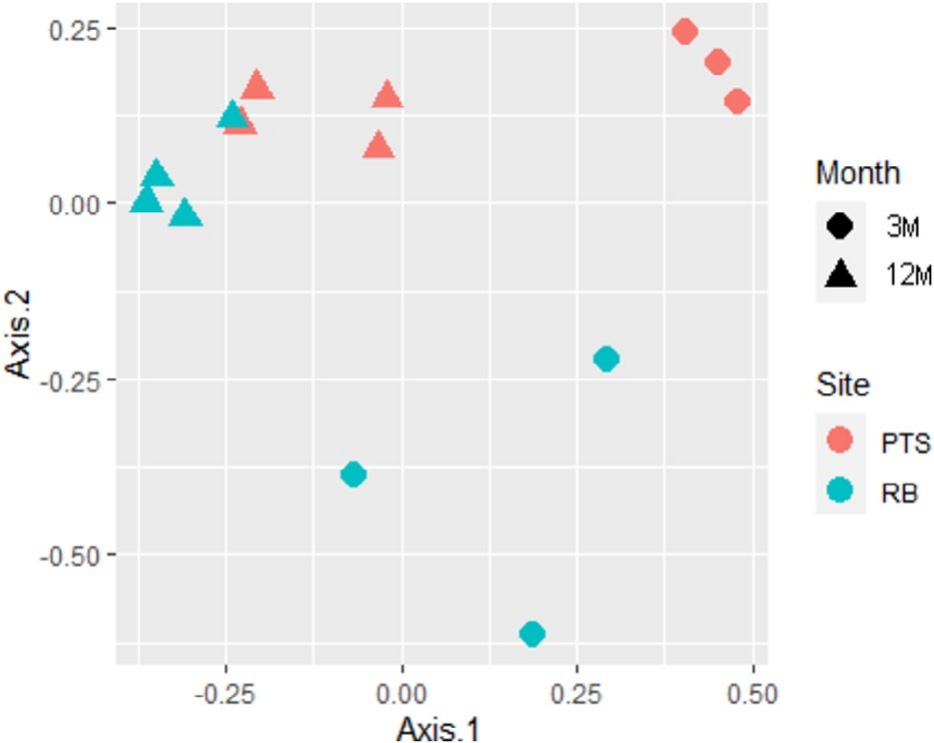
Beta‐diversity analysis on the Antarctic marine microbial biofilm communities collected in two different sites (RB or PTS) and at different colonisation times (3 or 12M). Principal coordinates analysis (PCoA) ordination plot based on Bray–Curtis dissimilarity index calculated on bacterial community composition data according to the variables site (RB or PTS) and/or colonisation time (month, 3 or 12M).

Richness of the biofilm samples (number of ZOTUs) was found to be significantly influenced by both the sampling site (RB, *z* value = 5.399, *p* < 0.001) and month (12M, *z* value = 102.517, *p* < 0.001) (Figure 3). In particular, biofilms collected after long‐term colonisation (12M) exhibited a significant increase in the number of ZOTUs detected per sample, indicating that the microbial marine biofilm communities in the early summer months were generally characterised by a few species which acted as pioneer colonisers of the new environment (i.e., PVC panel). Over time, the microbial communities continued to grow and develop, becoming more complex with the arrival of new species and genera, then finally establishing themselves by the end of the 12 months. Interestingly, either after the short‐ or long‐term colonisation, the impacted RB site microbial community appeared to be richer in the number of different bacterial species compared to the PTS one (ANOVA, month Chi^2^ = 11738.3, *p* < 0.001; site Chi^2^ = 22.9, *p* < 0.001) (Figure 3). As for beta diversity, 12M samples showed a close clustering in PCoA, highlighting the greater significance of the variable “month” with respect to the lower influence played by the variable “site” (Figure 4). On the contrary, early 3M samples composition appeared more variable (especially for RB ones), with clear differences displayed between the two sampling sites, in addition to their distinct separation from 12M samples (Figure 4). Microbial community composition was therefore confirmed to be shaped by both sampling month and site (PERMANOVA, site variable: mean squares = 0.68948, *F*. Model = 3.2210, *R* ^2^ = 0.15687, *p* = 0.002; month variable: mean squares = 1.06294, *F*. Model = 4.9657, *R* ^2^ = 0.24185, *p* = 0.001; combined site and month variables: mean squares = 0.50213, *F*. Model = 2.3458, *R* ^2^ = 0.11425, *p* = 0.011), even though seasonality appeared to exert a more significant effect. A general correlation of the microbial community composition with differences in the water physiochemical parameters and nutrient content was observed (Mantel test, *r* = 0.6365, *p* = 0.001) (Figure S1). The variance of these parameters also appeared strongly dependant on the “month” variable (PERMANOVA, Table S2). Thus, both richness and diversity of the Antarctic marine microbial biofilm communities followed the same trend, indicating that differences in the microbial communities depended mainly on the geographical site during the first period of colonisation (3M), evolving towards a stable and more homogeneous community composition after 12M of PVC panel immersion.

### 3.2 Composition and Relative Abundance of Antarctic Marine Microbial Biofilm Communities

**FIGURE 5:**
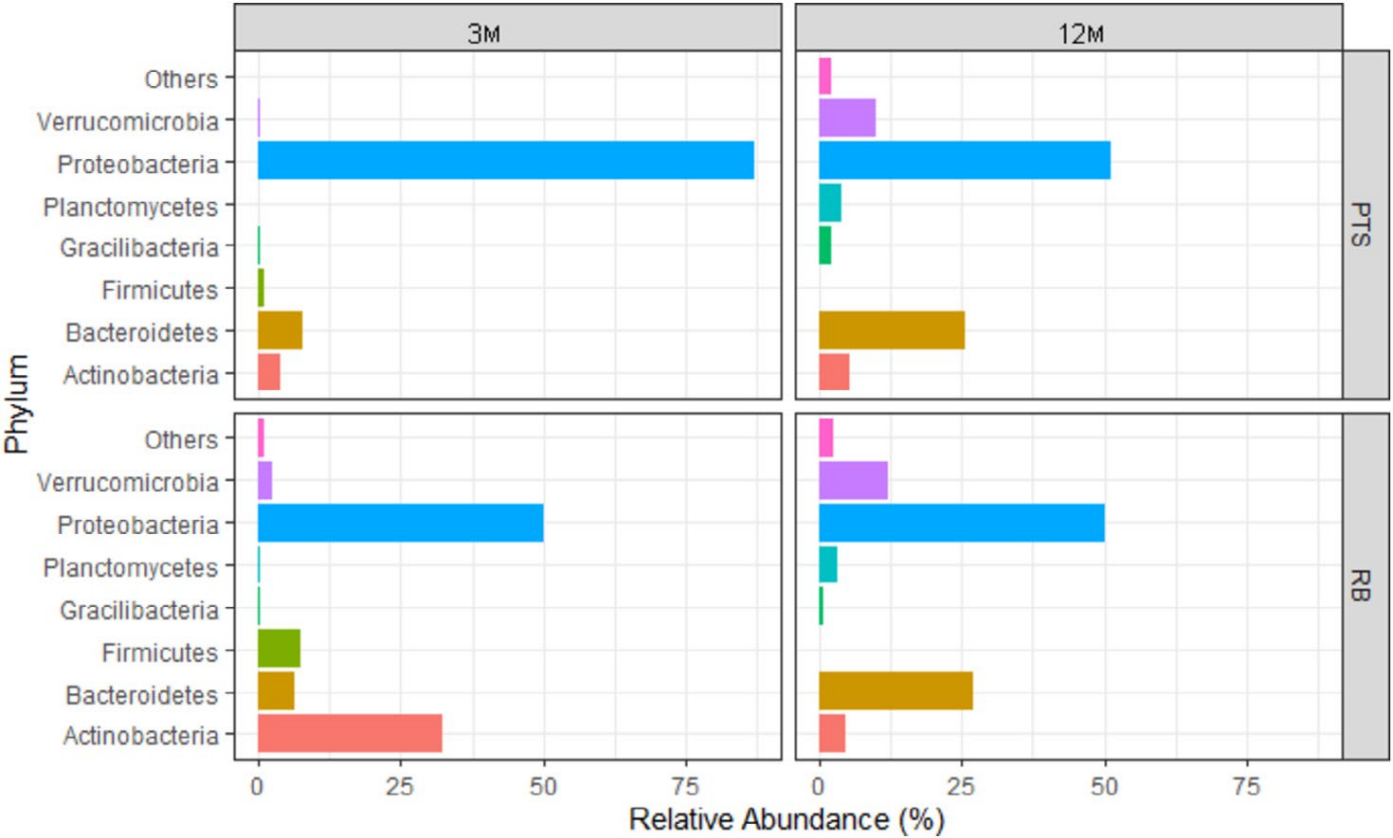
Taxonomic profiles of Antarctic marine microbial biofilm communities at phylum level, according to the site (RB or PTS) and colonisation time (3 or 12M). Others indicates the sum of the relative percentages of phyla that represented less than 0.5% of the total reads.

Overall, 95.44% of all the reads from 16S rRNA gene amplicon sequencing across the four types of Antarctic marine microbial biofilm samples were attributed to five bacterial phyla: *Proteobacteria* (55.85%), *Bacteroidetes* (18.07%), *Actinobacteria* (12.32%), *Verrucomicrobia* (7.07%) and *Firmicutes* (2.13%) (Figure S2). *Proteobacteria* was actually the predominant phylum in all types of samples, as it represented 50.27% of total reads in RB_3M, 50.36% in RB_12M, 51.06% in PTS_12M, and it was up to 87.15% in PTS_3M (Figure 5, Table S3). *Bacteroidetes* constituted 6.24% and 7.70% of total reads in RB_3M and PTS_3M and their presence increased up to 26.80% and 25.66% in 12M, respectively, while *Verrucomicrobia* represented 2.35% and 0.23% of total reads in RB_3M and PTS_3M, reaching 12.08% and 9.94% in RB_12M and PTS_12M, respectively (Figure 5, Table S3). *Actinobacteria* were uniquely abundant in RB_3M, where they were the second most abundant phylum at 32.29%, being instead less present in the other samples, representing 3.76%, 4.62% and 5.39% of total reads in PTS_3M, RB_12M and PTS_12M, respectively (Figure 5, Table S3). *Firmicutes* (7.24%) also appeared almost exclusively present in RB_3M samples. *Gracilibacteria* (from 0.08% in RB_3M up to 1.97% in PTS_12M) and *Planctomycetes* (from 0.05% in PTS_3M up to 3.74% in PTS_12M) were marginally present as well as a small group of other bacterial phyla, accounting for less < 0.5% of total reads (grouped together as Others, from 0.05% in PTS_3M up to 2.4% in RB_12M) (Figure 5, Table S3). The marine microbial biofilm communities were therefore dominated in all conditions by chemoorganotrophic taxa. These data further confirmed that the RB and PTS marine microbial biofilm communities displayed more marked differences after short‐term colonisation (in the middle of summertime), whereas they evolved towards a more similar composition with a prolonged colonisation period (12M) (Figure 5, Table S3).

**FIGURE 6:**
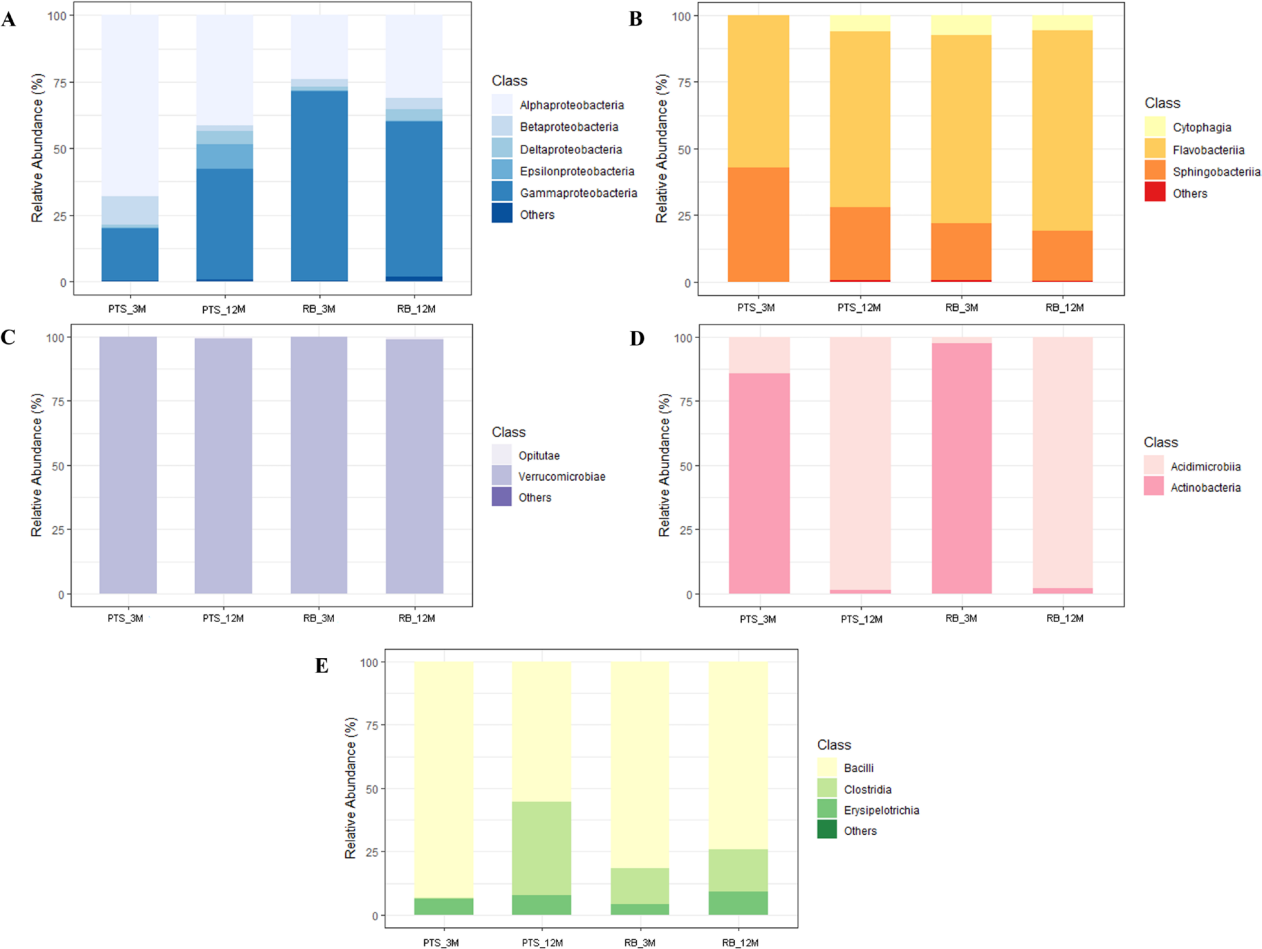
Relative composition of the most abundant bacterial phyla in Antarctic marine microbial biofilm communities at class level (PTS_3M, PTS_12M, RB_3M, RB_12M). Others indicates the sum of the relative percentages of classes that represented less than 0.5% of the total reads attributed to the phylum. (A) *Proteobacteria*. (B) *Bacteroidetes*. (C) *Verrucomicrobia*. (D) *Actinobacteria*. (E) *Firmicutes*.

At the class level, among *Proteobacteria*, *Alphaproteobacteria* were predominant in PTS samples (68.10% and 41.49% of total reads in 3 and 12M, respectively), while *Gammaproteobacteria* were most abundant in RB samples (70.91% and 58.08% in 3 and 12M, respectively) (Figure 6A, Table S4A). At the order level, *Rhodobacterales* was the most represented one belonging to *Proteobacteria* in PTS_3M (40.02%), PTS_12M (19.24%) and RB_12M (22.72%); in RB_3M, *Rhodobacterales* still constituted 16.61% of total reads, but *Pseudomonadales* largely dominated at 55.04% (Figure S3A, Table S4B). At the genus level, *Sulfitobacter* represented 22.49% of reads attributed to *Proteobacteria* in PTS_3M and *Psychrobacter* dominated at 45.73% in RB_3M, while a significant proportion of genera (26.22% in PTS and 30.93% in RB) remained unidentified in 12M samples, further highlighting the major complexity of the biofilm communities after 1‐year colonisation. A more detailed taxonomic analysis of the *Proteobacteria* community at the genus level can be found in Figure S3B and Table S4C.

Among *Bacteroidetes*, *Flavobacteriia* and *Flavobacteriales* were the most abundant class and order, respectively, in all types of samples (56.85% in PTS_3M, 66.06% in PTS_12M, 70.66% in RB_3M and 74.91% in RB_12M) (Figure 6B, Figure S4A, Table S5A,B). Between 21.85% and 37.99% of all *Bacteroidetes* reads could not be identified at the genus level, but *Crocinitomix* was the most common genus in PTS_3M (12.09%) and *Winogradskyella* in RB_3M (15.21%). They were both substituted by *Ulvibacter* in 12M samples (11.82% in PTS and 14.55% in RB) (Figure S4B, Table S5C). The class *Verrucomicrobiae* and the order *Verrucomicrobiales* represented between 98.87% and 100% of all detected reads for the *Verrucomicrobia* phylum (Figure 6C, Figure S5A, Table S6A,B), with *Rubritalea* as the predominant genus (from 31.07% in RB_12M up to 85.01% in PTS_3M) (Figure S5B, Table S6C).

Only two classes of *Actinobacteria* were identified through 16S rRNA gene amplicon sequencing and their distinct presence strongly correlated with the colonisation time of the biofilms (ANOVA, Sum sq. = 64.59, *F* = 14.9, *p* = 0.002). In fact, 85.70% and 97.50% of the *Actinobacteria* phylum total reads belonged to the *Actinobacteria* class in the PTS_3M and RB_3M samples, respectively, while 98.50% and 97.90% of the reads were instead attributed to the *Acidimicrobiia* class in the corresponding 12M samples (Figure 6D, Table S7A). Consequently, in 12M samples, the main order was *Acidimicrobiales* (98.49% in PTS and 97.92% in RB), while in 3M samples, most of the reads were attributed to either *Micrococcales* (64.57% in PTS) or *Propionibacteriales* (46.04% in RB) (Figure S6A, Table S7B). *Propionibacterium* (8.03% in PTS and 44.94% in RB) and *Microbacterium* (28.34% in RB and 41.63% in PTS) were the most abundant *Actinobacteria* genera in 3M, substituted by the *Acidimicrobiales* genus *Illumatobacter* (52.02% in PTS and 52.30% in RB) in 12M (Figure S6B, Table S7C). Finally, among *Firmicutes*, *Bacilli* (from 55.39% in PTS_12M up to 93.38% in PTS_3M) and *Bacillales* (from 41.54% in PTS_12M up to 74.33% in PTS_3M) were the most abundant class and order in all types of samples, respectively. *Lactobacillales* reached up to 19.05% in PTS_3M, while *Clostridia* and *Clostridiales* represented 32.31% and 36.92% of all *Firmicutes* reads in PTS_12M, respectively (Figure 6E, Figure S7A, Table S8A,B). The predominant *Firmicutes* genera in RB_3M were *Staphylococcus* (38.92%), *Bacillus* (20.11%) and *Anaerobacillus* (12.31%) (Figure S7B, Table S8C), whose presence was almost undetectable in the other samples.

### 3.3 Potential Pathobiome Analysis of the Antarctic Marine Microbial Biofilm Communities

**FIGURE 7:**
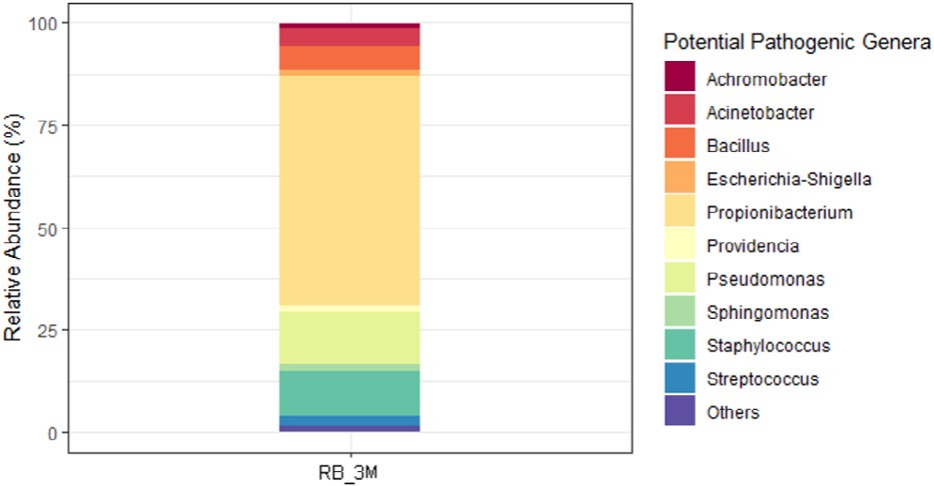
Relative abundance of the genera listed as potentially pathogenic (https://www.hartmann‐science‐center.com/en/hygiene‐knowledge/pathogens‐a‐z) in the marine microbial biofilm community from RB_3M samples. Others indicate the sum of the relative percentages of potential pathogenic bacterial genera that represented less than 0.5% of the total reads.

The presence and proportion of bacterial genera forming the potential pathobiome of an environment (Fox and Weisberg 2019), as they may harbour pathogenic species to humans, was also specifically investigated in the different biofilm samples. Their presence in Antarctic microbial biofilms could be linked to anthropogenic activities, such as superficial wastewater discharge practices. Consistently, in the 3M samples from the control site PTS, only 4.11% of identified reads could be attributed to the genera listed as potentially pathogenic, while from RB, their percentage significantly increased up to 27.49% (ANOVA, *p* < 0.001) (Table S9A), likely because of the closeness of this site to the wastewater treatment plant of the MZ research station. In Figure 7, the relative abundance of the different potentially pathogenic genera detected in RB_3M samples is shown, whereas their distribution across the different biofilm samples is reported in Table S9B and Figure S8. In RB_3M, 56.16% of reads were from the *Propionibacterium* genus, 13.04% from *Pseudomonas*, 11.00% from *Staphylococcus*, 5.69% from *Bacillus*, 4.76% from *Acinetobacter*, 2.40% from *Streptococcus*, 1.70% from *Sphingomonas*, 1.44% from *Escherichia–Shigella*, 1.22% from *Providencia* and 1.02% from *Achromobacter* (Figure 7). Interestingly, in a 1‐year period the two sites' distinct microbial biofilm communities did seem once more to converge towards a more similar composition, characterised by the nearly absolute presence of non‐pathogenic genera. Indeed, genera potentially pathogenic were practically absent in all 12M samples, either from RB or PTS samples, representing less than 0.4% of the total reads detected at the genus level (Table S9A,B).

### 3.4 Isolation of Cultivable Microorganisms From the Antarctic Marine Biofilm Samples

A total of 86 bacterial strains (75 Gram‐positive and 11 Gram‐negative) were collectively isolated and purified from all the marine biofilm samples applying selective enrichment procedures for spore‐forming and filamentous actinobacteria. A morphological dereplication step at both macroscopic and microscopic levels was introduced to limit re‐isolation of the same strains (see Experimental Procedures). The isolation media were added with antibiotics inhibiting fast‐growing Gram‐negative bacteria and fungi, and spore‐forming bacteria were enriched by heat treatment of the sample before the isolation procedure. Seven of the ten isolation media used had previously been optimised and adopted by other authors to specifically isolate filamentous actinobacteria, that is, Chitin agar, the five Isolation *Streptomyces* Project (ISP) agar and Oatmeal agar (Hsu and Lockwood 1975; Marcone et al. 2017). Moreover, different plates' incubation temperatures (i.e., 4°C, 20°C, and 28°C) were applied to try to capture as much bacterial diversity as possible, since many Antarctic marine microorganisms have been previously shown to be psychrotolerant rather than truly psychrophilic (Delille and Perret 1989; Pesciaroli et al. 2012).

Following these protocols, 50 strains were isolated in axenic culture from RB_3M, 25 strains from PTS_3M, 9 strains from RB_12M, and 2 strains from PTS_12M (Table S10). Indeed, all the isolates proved to be psychrotolerant, since even those isolated at 4°C were able to grow at 20°C; 19.77% of them was found to be strictly halophilic as they could not grow on their original isolation medium without the addition of artificial sea salt; the rest was anyhow halotolerant. The bacterial isolates belonged to 3 phyla distributed in 21 genera (Table 3): *Actinobacteria* was the most abundant phylum (54.65%), followed by *Firmicutes* (32.56%) and *Proteobacteria* (12.79%, *Gamma*‐ 10.47% and *Alpha*‐ 2.33%). Compared to the results of the culture‐independent approach, the predominance of Gram‐positive phyla, especially *Actinobacteria* (12.32% relative abundance from 16S rRNA gene amplicon sequencing, with *Firmicutes* at 2.13%), rather than *Proteobacteria* (55.85% from 16S rRNA gene amplicon sequencing), proved that the design of the selective culture conditions was effective and achieved its aim. At the genus level, the most abundant one was *Streptomyces* (22.09%), followed by *Arthrobacter* (10.47%), *Psychrobacter* (9.30%), *Sporosarcina* (8.14%), and *Bacillus* (5.81%), confirming that the isolation procedure mainly targeted filamentous actinobacteria as streptomycetes.

**TABLE 3:** Bacterial isolates (86) recovered from Antarctic marine biofilm samples through different selective culture conditions (detailed in Table S10).

Phylum	Genus	Strain ID number	Sample	Closest match	GenBank ID	Similarity (%)
*Firmicutes*	*Bacillus*	B3S	RB_3M	*Bacillus safensis* A3	MT890561.1	99.43
		B8S, B46	RB_3M, RB_3M	*Bacillus megaterium* Y2	MN509794.1	99.55
		B36S, B37S	RB_3M	*Bacillus australimaris* RSPG 10at	MT484119.1	99.28
	*Peribacillus*	B47S, B54S, B70S	PTS_3M, RB_3M	*Peribacillus frigoritolerans* AA11	MG571760.1	99.43
	*Lysinibacillus*	B254S	RB_12M	*Lysinibacillus xylanilyticus* PYK14	MF582342.1	99.57
	*Oceanobacillus*	B10S, B50S, B64S	PTS_3M, RB_3M	*Oceanobacillus rekensis* PT‐11	NR_145558.2	98.84
	*Planomicrobium*	B4S, B59S	RB_3M	*Planomicrobium okeanokoites* IHBB 9298	KR085812.1	99.88
	*Planococcus*	B84S, B87S	RB_3M	*Planococcus halocryophilus* DSM 24743	CP016537.2	99.43
	*Sporosarcina*	B6S, B12S	RB_3M, PTS_3M	*Sporosarcina psychrophila* DSM 6497	CP014616.1	99.57
		B67S, B85S, B86S	RB_3M	*Sporosarcina* sp. S11‐2	DQ514313.1	99.28
		B1, B45	RB_3M	*Sporosarcina globispora* IHBB 11049	KR085924.1	99.66
	*Paenisporosarcina*	B65S	PTS_3M	*Paenisporosarcina macmurdoensis* ES1‐24	KY012258.1	99.13
		B80S	RB_3M	*Paenisporosarcina* sp. PLR‐1‐1	MH018920.1	99.14
		B82S, B2	RB_3M	*Paenisporosarcina indica* PN2	NR_108473.1	99.00
	*Staphylococcus*	B71S	RB_3M	*Staphylococcus epidermidis* PB32	MN689679.1	99.57
*Actinobacteria*	*Rhodococcus*	B2S, B15S, B17S	RB_3M, PTS_3M	*Rhodococcus boritolerans* BTM‐1c	AB288061.1	99.86
	*Arthrobacter*	B13S, B69S	PTS_3M	*Arthrobacter* sp. R‐67793	KY386372.1	99.71
		B24S, B81S	RB_3M	*Arthrobacter flavus* A‐120	MF541537.1	99.56
		B43S, B83S	RB_3M	*Arthrobacter cheniae* B47	OQ130612.1	99.57
		B51S	PTS_3M	*Arthrobacter pascens* Z62	OQ130669.1	99.77
		B66S, B78S	PTS_3M, RB_3M	*Arthrobacter* sp. InS505	MN315438.1	99.57
	*Pseudoarthrobacter*	B75S, B248S	RB_3M, RB_12M	*Pseudarthrobacter oxydans* cqsm_g1	MN826535.1	99.18
		B249S	RB_12M	*Pseudarthrobacter* sp. genR30	MK641431.1	99.43
	*Plantibacter*	B11, B47	RB_3M	*Plantibacter flavus* cqsV15	MN826584.1	99.86
	*Brachybacterium*	B16, B21, B22, B32	RB_3M	*Brachybacterium* sp. 2.5	KF681045.1	99.42
	*Brevibacterium*	B44, B235S	RB_3M, PTS_12M	*Brevibacterium casei* FDAARGOS_990	CP065989.1	99.57
	*Micrococcus*	B20, B252S	RB_3M, RB_12M	*Micrococcus luteus* 3 L	KF993675.1	99.15
	*Dietzia*	B9S, B76S, B79S	RB_3M	*Dietzia psychralcaliphila* ILA‐1	CP015453.1	99.71
	*Streptomyces*	B18S, B28S	PTS_3M, RB_3M	*Streptomyces angustmyceticus* CGS‐B11	MZ317444.1	99.30
		B29S	RB_3M	*Streptomyces seoulensis* HEK131	AP025667.1	99.57
		B31S	RB_3M	*Streptomyces flavogriseus* F5	KU324434.1	99.72
		B32S	RB_3M	*Streptomyces recifensis* MMS19‐T31	MN658344.1	99.76
		B33S	RB_3M	*Streptomyces platensis* SAFN‐030	AY167807.1	99.43
		B34S, B255S	RB_3M, RB_12M	*Streptomyces fimicarius* A1	MK463974.1	99.72
		B38S	RB_3M	*Streptomyces microflavus* NA06532	CP054926.1	99.71
		B39S	PTS_3M	*Streptomyces cavourensis* NEAE‐42	KJ676478.1	99.53
		B40S	PTS_3M	*Streptomyces flavofuscus* CGMCC	JQ924407.1	99.57
		B42S	RB_3M	*Streptomyces anulatus* NBRC 15393	AB184644.1	99.71
		B44S	RB_3M	*Streptomyces caviscabies* ROA095	MZ026460.1	99.53
		B46S	PTS_3M	*Streptomyces pratensis* 1296_C11H	MK484235.1	99.76
		B48S	PTS_3M	*Streptomyces globisporus* BV	OQ195454.1	99.57
		B52S	PTS_3M	*Streptomyces* sp. LK‐R‐7	OQ456524.1	99.86
		B55S	PTS_3M	*Streptomyces* sp. AB368f	FR821241.1	99.29
		B60S	PTS_3M	*Streptomyces* sp. 111013air2	KP262511.1	99.16
		B62S	PTS_3M	*Streptomyces scabiei* OR9T	AB894410.1	99.57
*Gammaproteobacteria*	*Acinetobacter*	C33	RB_3M	*Acinetobacter pittii* 2012 N21‐164	CP033535.1	100.00
	*Psychrobacter*	B74S	PTS_3M	*Psychrobacter* sp. BIS1055	MN810234.1	99.71
		B34, B36	PTS_3M	*Psychrobacter glacincola* NBRC 101053	AB681354.1	99.88
		B236S	PTS_12M	*Psychrobacter* sp. AJ5	MF496375.1	99.57
		B247S, B17, B18	RB_3M, RB_12M	*Psychrobacter pacificensis* LPB0247	MH989594.1	98.79
		B253S	RB_12M	*Psychrobacter nivimaris* S2E6	MH362722.1	99.71
*Alphaproteobacteria*	*Paracoccus*	B250S	RB_12M	*Paracoccus lutimaris* HDM‐25	ON406416	99.11
		B251S	RB_12M	*Paracoccus gahaiensis* CUG00006	NR_178860.1	98.91

*Note:* Phylum and genus of the isolated strains identified through 16S rRNA gene sequencing, their closest phylogenetic match (and its corresponding GenBank ID) with the sequence similarity percentage detected by BLAST (basic local alignment search tool) are reported, as well as the sample of origin of the strains.

As detailed in Table S10, the highest degree of bacterial diversity was captured using the starch‐salts ISP4* (* additioned with 2% w/v artificial sea salt, see Table S1) as isolation medium, which yielded 34 isolates belonging to 15 different genera, including *Acinetobacter*, *Arthrobacter*, *Bacillus*, *Brevibacterium*, *Dietzia*, *Lysinibacillus*, *Oceanobacillus*, *Paenisporosarcina*, *Paracoccus*, *Peribacillus*, *Planomicrobium*, *Pseudoarthrobacter*, *Psychrobacter*, *Rhodococcus* and *Streptomyces*. Bacteria belonging to different genera (between 5 and 7) were also recovered on the culture media Marine Agar (10 strains), ISP2* (5 strains) and ISP6* (7 strains). *Planococcus* spp. were isolated only on minimal media such as ISP5* and ISP7*, while bacteria belonging to the *Brachybacterium* genus were originally isolated only from salt‐less media (even though they were then proved to tolerate high salt concentrations). Most of the strains were isolated at 20°C (66.28%), with 19.77% and 13.95% isolated at 28°C and 4°C, respectively. While the *Streptomyces* genus was almost exclusively retrieved at 20°C, bacteria belonging to *Arthrobacter*, *Bacillus*, *Paenisporosarcina*, *Planococcus*, *Plantibacter* and *Sporosarcina* genera were isolated at both 28°C and 4°C degrees too, denoting the wide adaptability to different temperatures of these strains. Biofilm samples that had been pretreated or diluted before plating yielded 26.74% and 24.42% of the isolates, respectively, but the diversity of bacterial genera thus recovered appeared similar to that from nontreated and nondiluted samples.

Regarding the geographical origin of the biofilms, isolates belonging to 19 different bacterial genera were retrieved from RB_3M samples (*Acinetobacter*, *Arthrobacter*, *Bacillus*, *Brachybacterium*, *Brevibacterium*, *Dietzia*, *Micrococcus*, *Oceanobacillus*, *Paenisporosarcina*, *Peribacillus*, *Planococcus*, *Planomicrobium*, *Plantibacter*, *Pseudoarthrobacter*, *Psychrobacter*, *Rhodococcus*, *Sporosarcina*, *Staphylococcus*, *Streptomyces*), 8 from PTS_3M (*Arthrobacter*, *Oceanobacillus*, *Paenisporosarcina*, *Peribacillus*, *Psychrobacter*, *Rhodococcus*, *Sporosarcina*, *Streptomyces*), 6 from RB_12M (*Lysinibacillus*, *Micrococcus*, *Paracoccus*, *Pseudoarthrobacter*, *Psychrobacter*, *Streptomyces*) and only 2 from PTS_12M (*Brevibacterium*, *Psychrobacter*). Thus, almost all the bacterial genera (21) identified through this culture‐dependent approach were isolated from the RB_3M samples. More genera were isolated from 3M samples in comparison to 12M ones; for example, *Streptomyces* and *Arthrobacter* genera were almost exclusively isolated in 3M, while *Psychrobacter* was the only genus recovered from all the biofilm samples. On the other hand, *Lysinibacillus* and *Paracoccus* genera were only isolated from RB_12M samples, while *Acinetobacter*, *Dietzia*, *Planococcus*, *Planomicrobium*, *Plantibacter* and *Staphylococcus* came exclusively from RB_3M. The results confirmed what emerged from the culture‐independent approach, that is, that the RB_3M biofilm was definitely the richest in Gram‐positive bacteria (either *Actinobacteria* or *Firmicutes*) and in potentially pathogenic genera, including *Staphylococcus* and *Acinetobacter* (Figures 5 and 7).

### 3.5 Screening of Bacterial Isolates From Antarctic Marine Biofilms for the Production of Hydrolytic and Oxidative Enzymes

**FIGURE 8:**
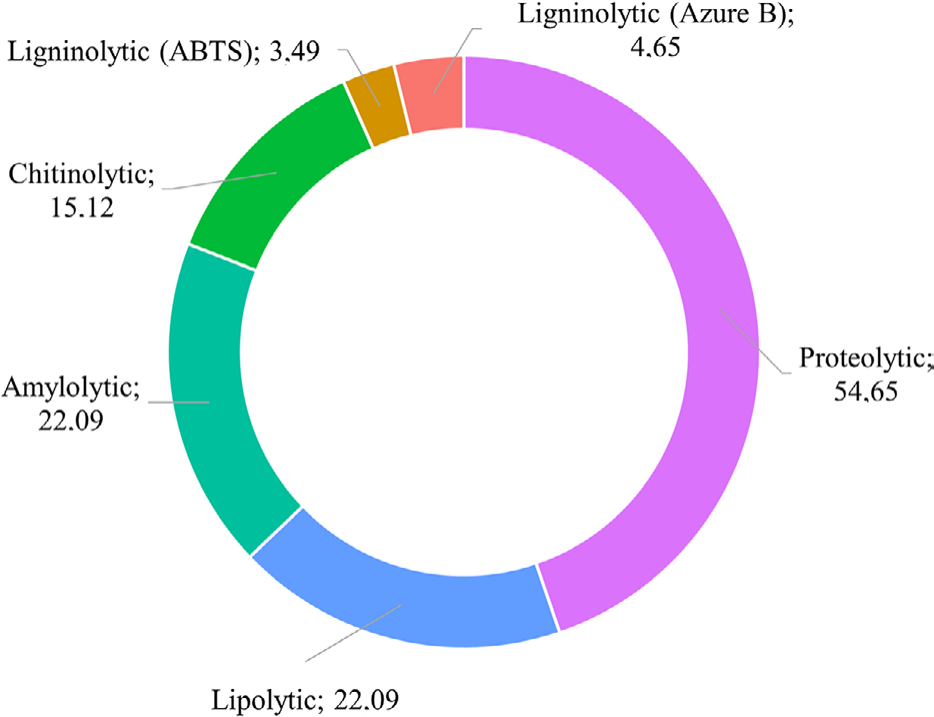
Distribution of hydrolytic and oxidative enzyme activities among the Antarctic bacterial isolates (86) from marine biofilms (expressed as percentage of strains possessing an enzyme activity on the total number of strains), detected by applying the degradation and/or colorimetric enzyme assays on agar plates described in Table 2.

As all the Antarctic marine biofilm isolates were found to be psychrotolerant, the enzyme activity screening was conducted at 20°C. It revealed that the most common enzyme activities among the isolates were the proteolytic ones (54.65% of the strains), followed by lipolytic and amylolytic (both 22.09%), chitinolytic (15.12%) and ligninolytic (4.65% on Azure B and 3.49% on ABTS) ones (Figure 8, Table S11). No cellulolytic activity was detected in any of the strains. At least one enzyme activity was produced by 68.60% of the strains, with some of them exhibiting multiple activities (25.58% two, 10.47% three and 2.33% four enzyme activities) (Table S11). Gram‐positive isolates were the best enzymes' producers, with 73.3% showing at least one enzyme activity, versus only 36.4% of the Gram‐negative isolates. *Arthrobacter*, *Bacillus*, *Oceanobacillus*, *Peribacillus*, *Pseudoarthrobacter* and *Streptomyces* were the more versatile genera exhibiting three to four different activities in the same strain (Table S11). Indeed, they were isolated on cultivation media, such as ISP4* and ISP2*, containing complex polymeric substrates, including starch and peptones, which can be slowly hydrolysed and assimilated by microorganisms due to their secretion of hydrolytic enzymes (Tables S1 and S10). These data confirm the potential of Antarctic marine *Actinobacteria* and *Firmicutes* species for bioprospecting purposes.

## 4 Discussion

In the last decades, the pristine environment of the Antarctic continent has been changing, threatened by several factors, including climate change and intensified anthropogenic activities, leading to the introduction of contaminants (e.g., heavy metals, antibiotics, microplastics) and allochthonous microorganisms, which may irreversibly modify native microbial communities (Lo Giudice et al. 2019; Cowan et al. 2011; Munari et al. 2017). Marine biofilms are considered hotspots of biodiversity, whose structure is subjected to rapid changes in response to substrate, salinity, temperature, nutrient content, ultraviolet radiation and water flux dynamics variations (Qian et al. 2022). In this research, we evaluated through a culture‐independent approach the microbial composition of biofilms colonising PVC panels submerged at −5 m in the RB and PTS sites in Terra Nova Bay over the course of 1 year (Figure 1). At both sites, the main environmental parameters showed similar seasonal fluctuations in terms of water temperature, pH, salinity, dissolved oxygen and nutrient content (Table 1), as described previously (Caroppo et al. 2022). Consistently, both richness and diversity of the marine biofilms were found to be influenced more by the length of the colonisation period (3 or 12M), rather than by the geographical site of origin. Indeed, early 3M biofilms showed a lower bacterial richness (higher in the impacted RB site compared to the pristine PTS), suggesting the presence of a few species acting as pioneer colonisers of the new environment. Anyhow, differences in bacterial community composition across sites were more marked in these 3M biofilms grown for a shorter colonisation time (Figures 3 and 4). On the contrary, samples from RB and PTS from 12M, despite the increased richness and complexity of their microbial communities, clustered more closely in PCoA than 3M ones, and shared a higher proportion of ZOTUs (3711 versus 1110 in 3M samples) (Figures 2 and 4). This indicates that over time the biofilms tend to stabilise and converge towards a more generalised homogeneous structure, irrespective of the site, as also highlighted by other studies on marine biofilms developing on stone seawalls in marine tropical waters (Summers et al. 2022), and plastic surfaces in Northern European and Northern Adriatic waters (Oberbeckmann et al. 2014; Pinto et al. 2019).

Regarding the taxonomic composition of the marine biofilms from Terra Nova Bay, *Proteobacteria* dominated the microbial communities in both 3 and 12M samples (Figure 5). *Alpha*‐ and *Gamma*‐ classes were the most abundant in PTS and RB, respectively (Figure 6), with *Rhodobacterales* as the most common order in almost all types of samples, except for RB_3M ones, where it was replaced by *Pseudomonadales*. Previous studies reported the predominance of *Alpha*‐ and *Gammaproteobacteria*, together with *Bacteroidetes*, in microbial communities associated to various kinds of plastics (e.g., PVC, low‐ and high‐density polyethylene, polypropylene, polyethylene terephthalate) in both tropical (Gomez and Onda 2022; Hernández‐Sánchez et al. 2023; Rajeev et al. 2019), temperate (Oberbeckmann et al. 2014; Pinto et al. 2019), and extreme marine environments (Papale et al. 2024; Webster and Negri 2006; Cappello et al. 2021; Pollet et al. 2018). For instance, Webster and Negri (Webster and Negri 2006) described *Alpha*‐ and *Gammaproteobacteria*, *Bacteroidetes*, and *Verrucomicrobia* as the predominant bacterial groups in a low‐impacted Antarctic site within McMurdo Sound. Additionally, both *Proteobacteria* and *Bacteroidetes* phyla were indicated as highly abundant among both the Antarctic and Arctic marine polar microbiota (Ghiglione et al. 2012; Cao et al. 2020). Conversely, in our marine biofilms, *Bacteroidetes* presence increased significantly along with *Verrucomicrobia* after 12M of colonisation but never became dominant (Figure 5). Anyhow, all these studies (including our work) confirmed the predominance of heterotrophic bacterial groups feeding on the organic matter produced by photoautotrophs (other prokaryotes, but mostly the eukaryotic *diatoms*), which are primarily found in sea ice and microbial mats in Antarctic aquatic environments (Wilkins et al. 2013). *Gammaproteobacteria* are known for their role in organic compounds degradation in extreme environments, as they are able to produce various hydrolytic extracellular enzymes (Yu et al. 2011; Yakimov et al. 2004). Among them, *Psychrobacter* spp. (abundant in RB_3M samples) can secrete cold‐adapted proteolytic enzymes, as well as degrade nitrobenzene and *p*‐nitrophenol (Denner et al. 2001; Wang et al. 2021). *Winogradskyella* and *Ulvibacter* spp., the most abundant *Bacteroidetes* genera in our Terra Nova Bay marine biofilms, are often found associated with marine invertebrates, algae, and spring bacterioplankton blooms (Jain and Krishnan 2017; Piontek et al. 2022), possessing carbohydrate degrading and extracellular polymeric substances secreting activities (Caruso et al. 2018; Choi et al. 2007). Indeed, the increase in their abundance in 12M samples was closely linked to a more preponderant presence of microalgae observed in Terra Nova Bay biofilm communities' structure after long‐term colonisation (Caroppo et al. 2022). Finally, in our marine biofilms, *Actinobacteria* and *Firmicutes* were present almost exclusively in RB_3M samples (Figure 5). *Propionibacterium* and *Microbacterium* were the most abundant *Actinobacteria* genera in 3M (together with *Staphylococcus* and *Bacillus* from *Firmicutes*). In 12M, they were replaced by *Illumatobacter*. These data agree with the results of previous metabarcoding studies that rarely identified *Actinobacteria* and *Firmicutes* among the main phyla characterising microbial biofilms from aquatic ecosystems (Signori et al. 2014; Papale et al. 2024; Lee et al. 2014; Li et al. 2019).

The presence in RB_3M samples of bacterial genera such as *Propionibacterium*, *Pseudomonas*, and *Staphylococcus* (Figure 7), which may harbour pathogenic species to humans (Dréno et al. 2018; Azam and Khan 2019; Otto 2009), strongly correlated with the anthropogenic impact of this site, due to its proximity to the wastewater treatment plant of the MZ research station, especially in the full summer season. Indeed, in summer, MZ research station is populated by scientists, while during the quieter winter period the released wastewater contaminating effect tends to be diluted, allowing the restoration of a more pristine microbial community, as observed in RB_12M samples. For example, for *Actinobacteria*, a marked shift from the potentially pathogenic *Propionibacteriales* order to the microalgae‐associated *Acidimicrobiales* one was observed between RB_3M and RB_12M samples. Similar findings were reported by other studies evaluating the efficacy of wastewater treatment systems in polar environments (Power et al. 2016; Huang et al. 2018).

By applying a culture‐dependent approach to the marine biofilm samples from Terra Nova Bay, our goal was to isolate mostly Gram‐positive microorganisms, especially filamentous *Actinobacteria*. These bacteria are well known for their extensive production of hydrolytic enzymes and bioactive metabolites; however, their potential in extreme environments, particularly in the Antarctic marine region, is still largely unexplored (Hui et al. 2021; Bull et al. 2005). The 86 bacterial strains (75 Gram‐positive and 11 Gram‐negative) we recovered belonged to three phyla: *Actinobacteria*, *Firmicutes* and *Proteobacteria*, distributed across 21 genera (Table 3). Eighteen of these genera were from the Gram‐positive branch, demonstrating that our targeted isolation procedure successfully enriched for this type of microorganisms, which were reported as relatively rare in the marine biofilm communities by the metabarcoding analysis. As indeed predicted by 16S rRNA gene amplicon sequencing, most of the Gram‐positive bacteria came from RB_3M samples (the richest in both *Actinobacteria* and *Firmicutes* sequences) (Figure 5). Nineteen of our 21 genera were isolated from RB_3M samples, including two strains belonging to *Staphylococcus* and *Acinetobacter* genera, and most closely related to the opportunistic pathogens *Staphylococcus epidermidis* and *Acinetobacter pittii* , further emphasising the anthropogenic impact on RB site during summer time (Table 3) (Fox and Weisberg 2019; Otto 2009; Weber, Harding, and Feldman 2015). Phylogenetically, the closest previously described related species of most of our isolates (i.e., from *Arthrobacter*, *Paenisporosarcina*, *Planomicrobium*, *Peribacillus*, *Psychrobacter* and *Streptomyces* genera) were isolated from the Antarctic environment (Reddy et al. 2000; Krishnamurthi et al. 2009; Saini et al. 2023; Heuchert et al. 2004; Su et al. 2013; Montecillo and Bae 2022) or they were anyhow members of genera identified from various cold extreme environments, including spacecraft assembly facilities and Arctic permafrost (Table 3) (Junge et al. 1998; Liu et al. 2000; Alam et al. 2003; Sun et al. 2023; Vipra et al. 2013; Shin et al. 2020; Tirumalai et al. 2018; Mykytczuk, Wilhelm, and Whyte 2012).

Compared to the number of genera (366) identified by metabarcoding, we isolated strains belonging to only 10 of them through the culture‐dependent approach, which represent the 2.73% of the total. Considering only the sequences corresponding to *Actinobacteria* and *Firmicutes* phyla, we isolated 7 genera out of the 55 detected by metabarcoding (12.73%) (Table S12). These results demonstrate that there is still room to improve cultivation‐based methods to better access the yet‐uncultivable microbial ‘dark matter’. On the other hand, we isolated bacteria belonging to 11 genera (i.e., *Brachybacterium*, *Dietzia*, *Lysinibacillus*, *Oceanobacillus*, *Paenisporosarcina*, *Peribacillus*, *Planomicrobium*, *Plantibacter*, *Pseudoarthrobacter*, *Sporosarcina* and *Streptomyces*) that we did not detect through 16S rRNA gene amplicon sequencing. Accordingly, Dziurzynski et al. (2023) reported that they could isolate 6.37% of the bacterial genera (57) identified by metabarcoding in the active layer of Arctic permafrost, and that 68 additional genera were retrieved only by cultivation. Similarly, in a study exploring the diversity of *Actinobacteria* in Arctic marine sediments, 10 genera were identified exclusively using cultivation techniques and 11 by molecular ones (Zhang et al. 2014). Such discrepancies could be due to limits in DNA extraction methods and polymerase chain reaction amplification, resulting in the underestimation of some genotypes from environmental samples (Baker, Smith, and Cowan 2003). In addition, selective culture conditions might enrich for rarer species, whose presence in the environment is normally below the detection level of molecular techniques or masked by the presence of highly abundant phylotypes. Overall, our results confirm how culture‐dependent and culture‐independent approaches might provide different complementary insights into the microbial diversity of environmental samples.

Finally, from the bioprospecting point of view, isolation of Antarctic marine bacteria is promising for the identification of novel valuable hydrolytic and oxidative enzyme activities, which may be advantageously integrated into current industrial processes, allowing to operate in more flexible environmental‐friendly conditions (Mangiagalli et al. 2020). For example, cold‐adapted proteases, lipases, amylases, chitinases and cellulases may be added to laundry detergents preparations or used as food additives and processing aids in the food industry. Cold‐ and halo‐tolerant laccases have shown promising properties for second‐ and third‐generation biofuels production or in highly contaminated water and soil bioremediation (Mesbah 2022; Hamid et al. 2022). Our Antarctic biofilm isolates were found to be psychrotolerant rather than truly psychrophilic, preferring to grow at temperatures near 20°C, which is consistent with what reported previously in several studies on Antarctic marine microorganisms' physiology (Delille and Perret 1989; Pesciaroli et al. 2012; Van Gestel, Ducklow, and Bååth 2020). Nevertheless, such psychrotolerant microbes have been proved to produce versatile cold‐ and halo‐tolerant enzymes which maintain high levels of activity at low temperatures (Fenice 2016; Bisaccia et al. 2023). The enzyme activity patterns we observed in the Antarctic isolates, with the prevalence of protease activities, followed by lipolytic and amylolytic ones (Figure 8), is consistent with proteins, nucleic acids, polysaccharides and lipids, representing the main organic components in marine biofilms. These compounds serve as energy source for microbial metabolism and play a central role in biofilm formation as part of the secreted extracellular polymeric substances matrix (Godwin Wesley and Satheesh 2009). Protease, lipase, and amylase activities were frequently detected in bacterial strains from different types of samples collected across maritime Antarctica, including sea, freshwater and ice (Loperena et al. 2012; Zhou et al. 2013; Lo Giudice et al. 2006). Chitinolytic and oxidoreductase activities were instead more rarely reported in Antarctic bacteria, whereas cellulase activities were found mostly in fungal strains (Loperena et al. 2012; Zhou et al. 2013; Lo Giudice et al. 2006, 2012; Araújo et al. 2011). In our screening (Table 2), multiple enzymes activities (≥ 3) were detected in strains belonging to *Arthrobacter*, *Bacillus*, *Oceanobacillus*, *Peribacillus* and *Streptomyces* genera, confirming the biotechnological potential of members of the *Actinobacteria* and *Firmicutes* phyla, as previously indicated by other authors (Loperena et al. 2012; Zhou et al. 2013; Lo Giudice et al. 2006, 2012; Araújo et al. 2011). Remarkably, *Oceanobacillus*, *Peribacillus* and *Streptomyces* genera, which produced up to four different enzyme activities per strain, were among those not detected by metabarcoding analyses, once more confirming the need to combine culture‐dependent and culture‐independent approaches for a comprehensive understanding of microbial diversity, even in a bioprospecting perspective.

In conclusion, our study contributed to characterising the biodiversity of marine microbial fouling in the Terra Nova Bay area, investigating bacterial biofilm communities and their shifts during a colonisation period of 1 year and in response to anthropogenic influence. Culture‐dependent and ‐independent approaches were compared, allowing to compensate for their respective limitations and draw an integrated and more complete picture of the marine microbial biofilm communities in this area of the Antarctic Ocean, as well as to get further insights into the potential future exploitation of their biotechnological potential.

## Author Contributions

**Bisaccia Melissa:** investigation, methodology, data curation, visualization, writing – original draft. **Binda Elisa:** conceptualization, supervision, visualization, writing – original draft. **Caruso Gabriella:** conceptualization, funding acquisition, project administration, writing – review and editing. **Azzaro Maurizio:** investigation, writing – review and editing. **Dell' Acqua Ombretta:** investigation, writing – review and editing. **Di Cesare Andrea:** software, methodology, data curation, writing – review and editing. **Ester Maria Eckert:** software, methodology, data curation, supervision, writing – review and editing. **Marinelli Flavia:** project administration, supervision, funding acquisition, conceptualization, writing – original draft, writing – review and editing.

## Conflicts of Interest

The authors declare no conflicts of interest.

## Supporting information


Appendix S1.

## Data Availability

16S rRNA gene amplicon sequencing data were submitted and are openly available in the Sequence Read Archive (SRA) of the National Center for Biotechnology Information (NCBI) under the BioProject accession number PRJNA1126177. 16S rRNA gene sequences from the Antarctic marine biofilm strains were deposited at GenBank (accession numbers PQ062283‐PQ062334).
