# Supplementary material for: Bacterial Diversity of Marine Biofilm Communities in Terra Nova Bay (Antarctica) by Culture‐Dependent and ‐Independent Approaches
Source: Environ Microbiol. 2025 Feb 2;27(2):e70045. doi: 10.1111/1462-2920.70045 (PMC11788576; doi:10.1111/1462-2920.70045)
Supplement: Supplementary file 1 — Appendix S1. [file EMI-27-e70045-s001.docx]

Appendix

**Table S1**

**Table S1.** Solid media used in the heterotrophic marine bacteria isolation procedure from Antarctic biofilm samples. Composition (in g/L) and commercial suppliers are reported. (*) indicates media which were also added with 20 g/L (2% w/v) artificial sea salt (Haquoss, Aquarialand, Turin, Italy). All the media components, unless otherwise stated, were obtained from Merck KGaA, (Darmstadt, Germany).

| **Medium** | **Composition (in g/L)** | **Reference** |
| --- | --- | --- |
| **Antarctic Bacterial Medium (ABM)** | 5 bacteriological peptone, 2 yeast extract, 20 agar | [1] |
| **Chitin Agar (*)** | 4 colloidal chitin, 0.7 K_2_HPO_4_, 0.5 MgSO_4_·7 H_2_O, 0.3 KH_2_PO_4_, 0.01 FeSO_4_, 0.001 ZnSO_4_, 0.001 MnCl_2_, 20 agar  Colloidal chitin was prepared from chitin from shrimp shells (Merck KGaA, Darmstadt, Germany) according to the protocol of Hsu et al., 1975 [42] | [2] |
| **Isolation Streptomyces Project agar (ISP) 2 (*)** | 10 dextrose, 5 bacteriological peptone, 3 yeast extract, 3 malt extract, 20 agar  (Himedia Laboratories, Mumbai, India) | [3] |
| **ISP4 (*)** | 10 soluble starch, 2 (NH_4_)_2_SO_4_, 2 CaCO_3_, 1 K_2_HPO_4,_ 1 MgSO_4_·7 H_2_O, 1 NaCl, 0.001 FeSO_4_·7 H_2_O, 0.001 MnCl_2_·7 H_2_O, 0.001 ZnSO_4_·7 H_2_O, 20 agar  (Himedia Laboratories, Mumbai, India) | [3] |
| **ISP5 (*)** | 1 L-asparagine, 1 K_2_HPO_4_, 10 mL/L glycerol, 1 mL/L Trace Elements Solution (0.001 FeSO_4_·7 H_2_O, 0.001 MnCl_2_·7 H_2_O, 0.001 ZnSO_4_·7 H_2_O), 20 agar  (Himedia Laboratories, Mumbai, India) | [3] |
| **ISP6 (*)** | 15 bacteriological peptone, 5 proteose peptone, 1 yeast extract, 1 K_2_HPO_4_, 0.5 ferric ammonium citrate, 0.08 sodium thiosulphate, 15 agar  (Himedia Laboratories, Mumbai, India) | [3] |
| **ISP7 (*)** | 1 L-asparagine, 0.5 L-tyrosine, 0.5 K_2_HPO_4_, 0.5 MgSO_4_·7 H_2_O, 0.5 NaCl, 15 mL/L glycerol, 1 mL/L Trace Elements Solution (in mg/L: 2.85 H_3_BO_3_, 1.8 MnCl_2_·4 H_2_O, 1.77 sodium tatrate, 1.36 FeSO_4_·7 H_2_O, 0.04 CoCl_2_·6 H_2_O, 0.027 CuCl_2_·2 H_2_O, 0.025 Na_2_MoO_4_·2 H_2_O, 0.02 ZnCl_2_), 20 agar  (Himedia Laboratories, Mumbai, India) | [3] |
| **Marine Agar** | 19.4 NaCl, 8.8 MgCl_2_, 5 bacteriological peptone, 3.24 Na_2_SO_4_, 1.8 CaCl_2_, 1 yeast extract, 0.55 KCl, 0.16 NaHCO_3_, 0.1 ferric citrate, 0.08 KBr, 0.022 H_3_BO_3_, 0.008 Na_2_HPO_4_ 0.004 sodium silicate, 0.0034 SrCl_2_, 0.0024 NaF, 0.0016 NH_4_NO_3_, 15 agar  (Conda Laboratories, Madrid, Spain) | [4] |
| **Oatmeal Agar** | 20 oatmeal, 20 agar | [3] |
| **Seawater Agar** | 20 artificial sea salt, 15 agar | [5] |

**Figure S1**


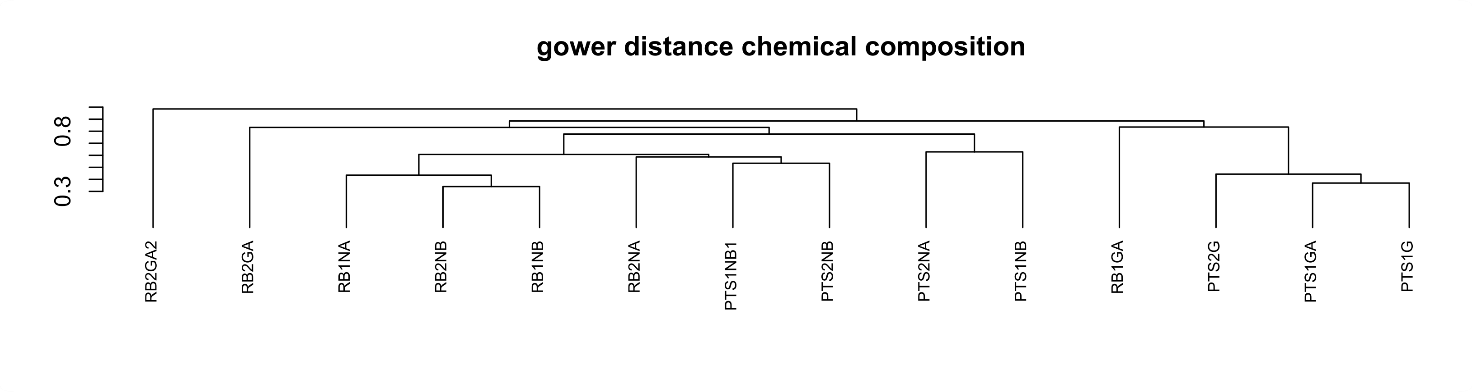
**Figure S1.** Antarctic marine microbial biofilm samples clustering on the basis of Gower’s distance (calculated on water physiochemical parameters and nutrient content at sampling site and colonization time).

**Table S2**

**Table S2.** PERMANOVA (permutational multivariate analysis of variance) analysis evaluating the effect of sampling site and colonization time (month) on Gower’s distances (calculated on water physiochemical parameters and nutrient content at sampling site and colonization time).

|  | **Df** | **SumOfSqs** | **R^2^** | **F** | ***p*** |  |
| --- | --- | --- | --- | --- | --- | --- |
| Site | 1 | 0.04048 | 0.02083 | 6,59E+19 | 0.001 | *** |
| Month | 1 | 156.555 | 0.80574 | 2,55E+21 | 0.001 | *** |
| Site:Month | 1 | 0.33696 | 0.17342 | 5,49E+20 | 0.001 | *** |
| Residual | 10 | 0.00000 | 0.00000 |  |  |  |
| Total | 13 | 194.299 | 100.000 |  |  |  |

**Figure S2**


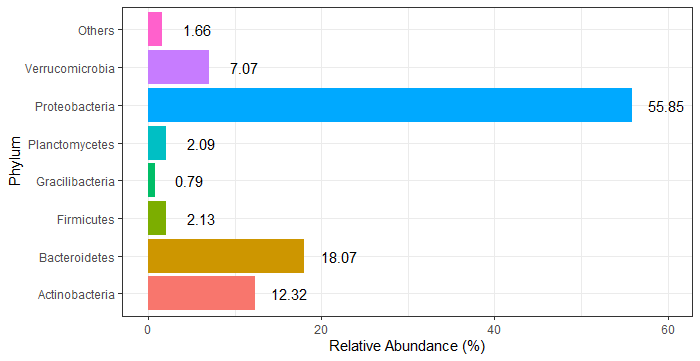


**Figure S2.** Global taxonomic profile at phylum level of the Antarctic marine microbial biofilm communities under investigation in this study. Others indicates the sum of the relative percentages of phyla that represented less than 0.5% of the total reads.

**Table S3**

**Table S3.** Relative abundance (%) of the phyla in the Antarctic marine microbial biofilm communities (PTS_3M, PTS_12M, RB_3M, RB_12M, where PTS indicates Punta Stocchino, RB indicates Road Bay, 3M indicates three months and 12M indicates twelve months). Others indicate the sum of the relative percentages of phyla that represented less than 0.5% of the total reads.

| **Phylum** | **PTS_3M**  **(%)** | **PTS_12M**  **(%)** | **RB_3M**  **(%)** | **RB_12M**  **(%)** |
| --- | --- | --- | --- | --- |
| *Actinobacteria* | 3.76 | 5.39 | 32.29 | 4.62 |
| *Bacteroidetes* | 7.70 | 25.66 | 6.24 | 26.80 |
| *Firmicutes* | 0.88 | 0.02 | 7.24 | 0.02 |
| *Gracilibacteria* | 0.16 | 1.97 | 0.08 | 0.59 |
| *Planctomycetes* | 0.05 | 3.74 | 0.38 | 3.11 |
| *Proteobacteria* | 87.15 | 51.06 | 50.27 | 50.36 |
| *Verrucomicrobia* | 0.23 | 9.94 | 2.35 | 12.08 |
| Others | 0.05 | 2.21 | 1.14 | 2.41 |

**Tables S4A, S4B, S4C**

**Table S4A.** Relative abundance (%) at class level of Proteobacteria in the Antarctic marine microbial biofilm communities (PTS_3M, PTS_12M, RB_3M, RB_12M). Others indicate the sum of the relative percentages of classes that represented less than 0.5% of the total reads attributed to the phylum. Unidentified indicates the relative percentage of classes which could not be identified to the corresponding taxonomic level.

| **Proteobacteria_Class** | **PTS_3M**  **(%)** | **PTS_12M**  **(%)** | **RB_3M**  **(%)** | **RB_12M**  **(%)** |
| --- | --- | --- | --- | --- |
| *Alpha* | 68.10 | 41.49 | 24.11 | 31.15 |
| *Beta* | 10.80 | 1.93 | 3.02 | 4.38 |
| *Delta* | 1.10 | 4.95 | 1.14 | 4.43 |
| *Epsilon* | 0.40 | 9.48 | 0.63 | 0.30 |
| *Gamma* | 19.32 | 41.61 | 70.91 | 58.08 |
| Others | 0.40 | 0.54 | 0.44 | 1.84 |

**Table S4B.** Relative abundance (%) at order level of Proteobacteria in the Antarctic marine microbial biofilm communities (PTS_3M, PTS_12M, RB_3M, RB_12M). Others indicate the sum of the relative percentages of orders that represented less than 0.5% of the total reads attributed to the phylum. Unidentified indicates the relative percentage of orders which could not be identified to the corresponding taxonomic level.

| **Proteobacteria_Order** | **PTS_3M**  **(%)** | **PTS_12M**  **(%)** | **RB_3M**  **(%)** | **RB_12M**  **(%)** |
| --- | --- | --- | --- | --- |
| Unidentified | 0.40 | 0.47 | 0.44 | 1.84 |
| *Alteromonadales* | 0.73 | 8.32 | 2.25 | 15.84 |
| *Arenicellales* | 0.08 | 1.18 | 0.24 | 3.04 |
| *BD7-8_marine_group* | 0.16 | 7.16 | 0.29 | 4.38 |
| *Bdellovibrionales* | 0.98 | 1.31 | 0.70 | 1.02 |
| *Campylobacterales* | 0.40 | 9.48 | 0.53 | 0.30 |
| *Caulobacterales* | 1.85 | 6.32 | 0.53 | 1.45 |
| *Cellvibrionales* | 1.83 | 9.22 | 2.37 | 14.14 |
| *Chromatiales* | 9.98 | 1.39 | 0.77 | 2.27 |
| *Desulfobacterales* | 0.00 | 1.35 | 0.23 | 1.35 |
| *E01-9C-26_marine_group* | 0.01 | 1.27 | 0.63 | 1.99 |
| *Methylophilales* | 10.63 | 1.71 | 0.87 | 4.10 |
| *Myxococcales* | 0.04 | 1.02 | 0.17 | 0.95 |
| *Oceanospirillales* | 2.21 | 3.40 | 3.68 | 2.54 |
| *Order_Incertae_Sedis* | 0.01 | 1.35 | 0.54 | 4.26 |
| *Pseudomonadales* | 1.26 | 0.07 | 55.04 | 0.04 |
| *Rhizobiales* | 21.54 | 8.38 | 4.35 | 2.78 |
| *Rhodobacterales* | 40.02 | 19.24 | 16.61 | 22.72 |
| *Sphingomonadales* | 3.81 | 6.47 | 1.58 | 2.74 |
| *Thiotrichales* | 2.41 | 6.82 | 0.49 | 2.59 |
| *Xanthomonadales* | 0.01 | 0.87 | 2.36 | 5.76 |
| Others | 1.62 | 3.21 | 5.31 | 3.92 |

**Table S4C.** Relative abundance (%) at genus level of Proteobacteria in the Antarctic marine microbial biofilm communities (PTS_3M, PTS_12M, RB_3M, RB_12M). Others indicate the sum of the relative percentages of genera that represented less than 0.5% of the total reads attributed to the phylum. Unidentified indicates the relative percentage of genera which could not be identified to the corresponding taxonomic level.

| **Proteobacteria_Genus** | **PTS_3M**  **(%)** | **PTS_12M**  **(%)** | **RB_3M**  **(%)** | **RB_12M**  **(%)** |
| --- | --- | --- | --- | --- |
| Unidentified | 8.05 | 26.22 | 6.51 | 30.93 |
| *Acinetobacter* | 0.20 | 0.01 | 2.44 | 0.02 |
| *Algimonas* | 0.43 | 1.04 | 0.01 | 0.75 |
| *Arenicella* | 0.08 | 1.09 | 0.24 | 2.40 |
| *BD1-7_clade* | 0.78 | 0.51 | 0.12 | 1.12 |
| *Cocleimonas* | 0.12 | 2.85 | 0.16 | 2.01 |
| *Colwellia* | 0.24 | 3.54 | 0.56 | 1.86 |
| *Erythrobacter* | 0.33 | 0.83 | 0.34 | 0.73 |
| *Glaciecola* | 0.04 | 1.41 | 0.09 | 4.39 |
| *Granulosicoccus* | 4.98 | 1.16 | 0.72 | 1.68 |
| *Haliea* | 0.08 | 0.86 | 0.11 | 1.58 |
| *Halioglobus* | 0.76 | 2.06 | 0.94 | 4.26 |
| *Halomonas* | 0.13 | 0.00 | 3.35 | 0.00 |
| *Hellea* | 0.72 | 1.32 | 0.01 | 0.06 |
| *Hoeflea* | 9.21 | 0.92 | 0.11 | 0.40 |
| *Kangiella* | 0.76 | 0.87 | 0.05 | 1.04 |
| *Loktanella* | 1.18 | 0.86 | 0.66 | 2.49 |
| *Marinicella* | 0.01 | 1.35 | 0.54 | 4.26 |
| *Mesorhizobium* | 2.09 | 0.28 | 0.01 | 0.09 |
| *Methylotenera* | 10.63 | 1.71 | 0.85 | 4.06 |
| *Moritella* | 0.05 | 0.79 | 1.59 | 0.91 |
| *OM27_clade* | 0.98 | 0.94 | 0.36 | 0.29 |
| *OM60[NOR5]_clade* | 0.10 | 2.92 | 0.38 | 3.00 |
| *Paraglaciecola* | 0.38 | 1.82 | 0.00 | 1.17 |
| *Planktotalea* | 4.90 | 3.38 | 0.45 | 0.13 |
| *Pseudahrensia* | 6.57 | 5.94 | 0.37 | 1.64 |
| *Pseudomonas* | 1.01 | 0.05 | 6.68 | 0.03 |
| *Psychrobacter* | 0.00 | 0.00 | 45.73 | 0.00 |
| *Robiginitomaculum* | 0.15 | 3.52 | 0.05 | 0.26 |
| *Roseobacter* | 2.18 | 0.38 | 0.94 | 1.01 |
| *Roseobacter_clade_NAC11-7_lineage* | 0.47 | 3.09 | 0.24 | 1.71 |
| *Shewanella* | 0.00 | 0.35 | 0.00 | 7.35 |
| *Sphingomonas* | 1.55 | 0.28 | 0.87 | 0.08 |
| *Sphingorhabdus* | 1.55 | 3.55 | 0.23 | 1.32 |
| *Sulfitobacter* | 22.49 | 2.88 | 9.10 | 5.21 |
| *Sulfurimonas* | 0.38 | 7.66 | 0.51 | 0.16 |
| *Thiorhodospira* | 4.99 | 0.19 | 0.03 | 0.15 |
| Others | 11.43 | 13.33 | 14.60 | 11.45 |

**Figures S3A, S3B**


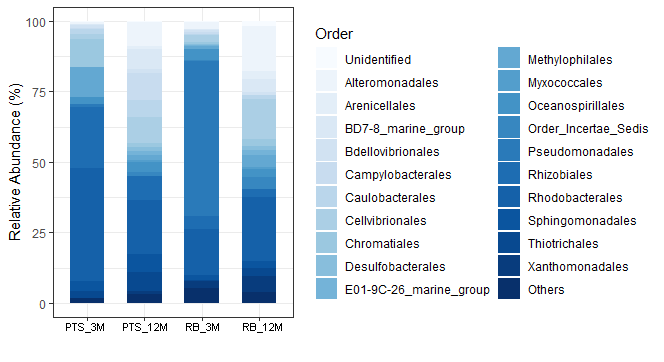

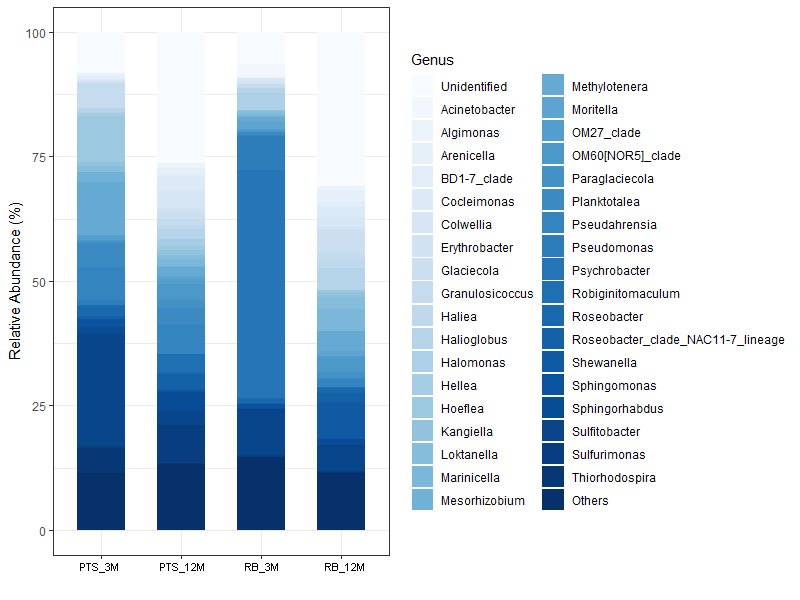


**A**

**B**

**Figure S3.** Relative abundance (%) at order **(A)** and genus **(B)** level of Proteobacteria in the Antarctic marine microbial biofilm communities (PTS_3M, PTS_12M, RB_3M, RB_12M). Others indicate the sum of the relative percentages of orders or genera that represented less than 0.5% of the total reads attributed to the phylum. Unidentified indicates the relative percentage of orders or genera which could not be identified to the corresponding taxonomic level.

**Table S5A, S5B, S5C**

**Table S5A.** Relative abundance (%) at class level of Bacteroidetes in the Antarctic marine microbial biofilm communities (PTS_3M, PTS_12M, RB_3M, RB_12M). Others indicate the sum of the relative percentages of classes that represented less than 0.5% of the total reads attributed to the phylum. Unidentified indicates the relative percentage of classes which could not be identified to the corresponding taxonomic level.

| **Bacteroidetes_Class** | **PTS_3M**  **(%)** | **PTS_12M**  **(%)** | **RB_3M**  **(%)** | **RB_12M**  **(%)** |
| --- | --- | --- | --- | --- |
| *Cytophagia* | 0.15 | 6.10 | 7.39 | 5.84 |
| *Flavobacteriia* | 56.85 | 66.06 | 70.66 | 74.91 |
| *Sphingobacteriia* | 43.00 | 27.18 | 21.37 | 18.80 |
| Others | 0.00 | 0.64 | 0.57 | 0.45 |

**Table S5B.** Relative abundance (%) at order level of Bacteroidetes in the Antarctic marine microbial biofilm communities (PTS_3M, PTS_12M, RB_3M, RB_12M). Others indicate the sum of the relative percentages of orders that represented less than 0.5% of the total reads attributed to the phylum. Unidentified indicates the relative percentage of orders which could not be identified to the corresponding taxonomic level.

| **Bacteroidetes_Order** | **PTS_3M**  **(%)** | **PTS_12M**  **(%)** | **RB_3M**  **(%)** | **RB_12M**  **(%)** |
| --- | --- | --- | --- | --- |
| Unidentified | 0.00 | 0.05 | 0.24 | 0.22 |
| *Cytophagales* | 0.15 | 6.04 | 5.55 | 4.93 |
| *Flavobacteriales* | 56.85 | 66.06 | 70.66 | 74.91 |
| *Order_II* | 0.00 | 0.07 | 1.84 | 0.91 |
| *Sphingobacteriales* | 43.00 | 27.18 | 21.37 | 18.80 |
| Others | 0.00 | 0.60 | 0.34 | 0.22 |

**Table S5C.** Relative abundance (%) at genera level of Bacteroidetes in the Antarctic marine microbial biofilm communities (PTS_3M, PTS_12M, RB_3M, RB_12M). Others indicate the sum of the relative percentages of genera that represented less than 0.5% of the total reads attributed to the phylum. Unidentified indicates the relative percentage of genera which could not be identified to the corresponding taxonomic level.

| **Bacteroidetes_Genus** | **PTS_3M**  **(%)** | **PTS_12M**  **(%)** | **RB_3M**  **(%)** | **RB_12M**  **(%)** |
| --- | --- | --- | --- | --- |
| Unidentified | 37.99 | 33.31 | 30.33 | 21.85 |
| *Algibacter* | 0.94 | 3.64 | 1.87 | 2.51 |
| *Aquibacter* | 0.64 | 1.31 | 0.81 | 1.42 |
| *Crocinitomix* | 12.09 | 3.15 | 0.01 | 0.85 |
| *Cryomorpha* | 0.24 | 0.68 | 0.64 | 1.45 |
| *Fluviicola* | 7.76 | 2.97 | 0.03 | 0.94 |
| *Fulvivirga* | 0.04 | 1.00 | 0.12 | 1.30 |
| *Kordia* | 0.13 | 0.93 | 0.00 | 0.51 |
| *Lewinella* | 9.82 | 3.86 | 1.15 | 3.28 |
| *Lutibacter* | 0.02 | 1.77 | 0.01 | 0.76 |
| *Lutimonas* | 0.01 | 0.97 | 0.87 | 2.52 |
| *Maribacter* | 0.15 | 3.59 | 2.15 | 4.25 |
| *Maritimimonas* | 0.14 | 2.52 | 4.38 | 3.35 |
| *Mesoflavibacter* | 0.06 | 0.50 | 0.50 | 0.64 |
| *Olleya* | 0.42 | 0.93 | 0.92 | 0.97 |
| *Owenweeksia* | 1.21 | 3.86 | 3.83 | 3.21 |
| *Pibocella* | 0.65 | 2.30 | 0.65 | 2.34 |
| *Polaribacter* | 9.34 | 4.39 | 1.57 | 3.87 |
| *Portibacter* | 0.47 | 0.81 | 0.46 | 1.74 |
| *Pricia* | 0.16 | 0.14 | 5.88 | 3.36 |
| *Psychroserpens* | 3.15 | 1.86 | 1.19 | 2.48 |
| *Reichenbachiella* | 0.00 | 0.80 | 0.00 | 1.17 |
| *Rubidimonas* | 0.45 | 1.33 | 0.00 | 0.40 |
| *Schleiferia* | 0.04 | 0.71 | 1.57 | 2.15 |
| *Taibaiella* | 0.00 | 0.49 | 2.13 | 1.00 |
| *Ulvibacter* | 1.62 | 11.82 | 10.98 | 14.55 |
| *Wenyingzhuangia* | 5.73 | 0.87 | 0.31 | 2.09 |
| *Winogradskyella* | 0.76 | 4.76 | 15.21 | 10.10 |
| Others | 5.96 | 4.74 | 12.45 | 4.90 |

**Figures S4A, S4B**


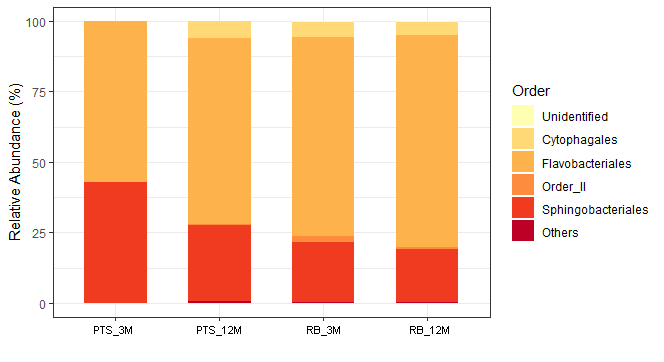

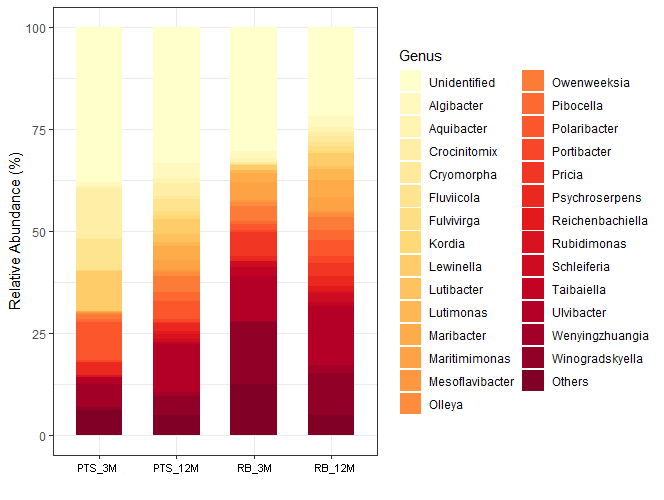


**A**

**B**

**Figure S4.** Relative abundance (%) at order **(A)** and genus **(B)** level of Bacteroidetes in the Antarctic marine microbial biofilm communities (PTS_3M, PTS_12M, RB_3M, RB_12M). Others indicate the sum of the relative percentages of orders or genera that represented less than 0.5% of the total reads attributed to the phylum. Unidentified indicates the relative percentage of orders or genera which could not be identified to the corresponding taxonomic level.

**Table S6A, S6B, S6C**

**Table S6A.** Relative abundance (%) at class level of Verrucomicrobia in the Antarctic marine microbial communities (PTS_3M, PTS_12M, RB_3M, RB_12M). Others indicate the sum of the relative percentages of classes that represented less than 0.5% of the total reads attributed to the phylum.

| **Verrucomicrobia_Class** | **PTS_3M**  **(%)** | **PTS_12M**  **(%)** | **RB_3M**  **(%)** | **RB_12M**  **(%)** |
| --- | --- | --- | --- | --- |
| *Opitutae* | 0.00 | 0.80 | 0.02 | 1.12 |
| *Verrucomicrobiae* | 100.00 | 99.20 | 99.96 | 98.87 |
| Others | 0.00 | 0.00 | 0.02 | 0.01 |

**Table S6B.** Relative abundance (%) at order level of Verrucomicrobia in the Antarctic marine microbial communities (PTS_3M, PTS_12M, RB_3M, RB_12M). Others indicate the sum of the relative percentages of orders that represented less than 0.5% of the total reads attributed to the phylum.

| **Verrucomicrobia_Order** | **PTS_3M**  **(%)** | **PTS_12M**  **(%)** | **RB_3M**  **(%)** | **RB_12M**  **(%)** |
| --- | --- | --- | --- | --- |
| *Opitutales* | 0.00 | 0.80 | 0.02 | 1.12 |
| *Verrucomicrobiales* | 100.00 | 99.20 | 99.96 | 98.87 |
| Others | 0.00 | 0.00 | 0.02 | 0.01 |

**Table S6C.** Relative abundance (%) at genus level of Verrucomicrobia in the Antarctic marine microbial communities (PTS_3M, PTS_12M, RB_3M, RB_12M). Others indicate the sum of the relative percentages of genera that represented less than 0.5% of the total reads attributed to the phylum. Unidentified indicates the relative percentage of genera which could not be identified to the corresponding taxonomic level.

| **Verrucomicrobia_Genus** | **PTS_3M**  **(%)** | **PTS_12M**  **(%)** | **RB_3M**  **(%)** | **RB_12M**  **(%)** |
| --- | --- | --- | --- | --- |
| Unidentified | 2.10 | 14.85 | 26.48 | 23.96 |
| *Haloferula* | 0.70 | 1.32 | 17.43 | 6.07 |
| *Luteolibacter* | 0.35 | 0.11 | 0.02 | 1.02 |
| *Persicirhabdus* | 2.79 | 5.86 | 2.87 | 17.59 |
| *Roseibacillus* | 9.06 | 5.04 | 10.80 | 19.14 |
| *Rubritalea* | 85.01 | 71.97 | 41.52 | 31.07 |
| Others | 0.00 | 0.85 | 0.89 | 1.15 |

**Figures S5A, S5B**


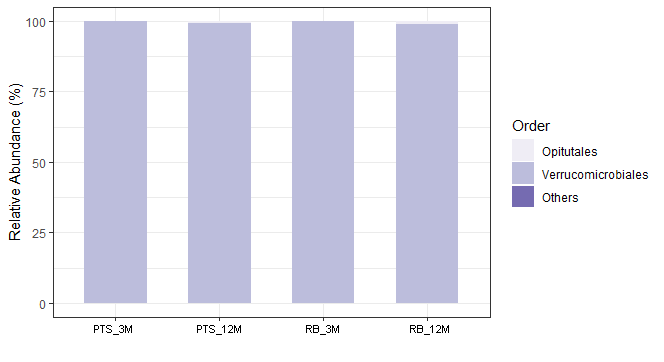

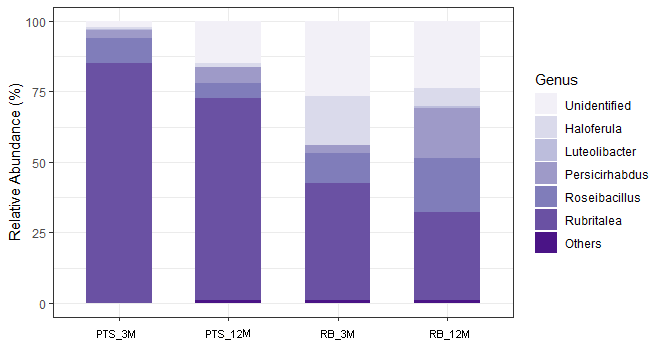


**A**

**B**

**Figure S5.** Relative abundance (%) at order **(A)** and genus **(B)** level of Verrucomicrobia in the Antarctic marine microbial biofilm communities (PTS_3M, PTS_12M, RB_3M, RB_12M). Others indicate the sum of the relative percentages of orders or genera that represented less than 0.5% of the total reads attributed to the phylum. Unidentified indicates the relative percentage of orders or genera which could not be identified to the corresponding taxonomic level.

**Table S7A, S7B, S7C**

**Table S7A.** Relative abundance (%) at class level of Actinobacteria in the Antarctic marine microbial biofilm communities (PTS_3M, PTS_12M, RB_3M, RB_12M).

| **Actinobacteria_Class** | **PTS_3M**  **(%)** | **PTS_12M**  **(%)** | **RB_3M**  **(%)** | **RB_12M**  **(%)** |
| --- | --- | --- | --- | --- |
| *Acidimicrobiia* | 14.2 | 98.50 | 2.50 | 97.90 |
| *Actinobacteria* | 85.8 | 1.50 | 97.50 | 2.10 |

**Table S7B.** Relative abundance (%) at order level of Actinobacteria in the Antarctic marine microbial biofilm communities (PTS_3M, PTS_12M, RB_3M, RB_12M). Others indicate the sum of the relative percentages of orders that represented less than 0.5% of the total reads attributed to the phylum.

| **Actinobacteria_Order** | **PTS_3M**  **(%)** | **PTS_12M**  **(%)** | **RB_3M**  **(%)** | **RB_12M**  **(%)** |
| --- | --- | --- | --- | --- |
| *Acidimicrobiales* | 14.24 | 98.49 | 2.48 | 97.92 |
| *Corynebacteriales* | 6.68 | 0.15 | 8.37 | 0.09 |
| *Micrococcales* | 64.57 | 0.52 | 42.67 | 1.67 |
| *Propionibacteriales* | 8.82 | 0.84 | 46.04 | 0.32 |
| Others | 5.68 | 0.00 | 0.43 | 0.00 |

**Supplementary Table S7C.** Relative abundance (%) at genus level of Actinobacteria in the Antarctic marine microbial biofilm communities (PTS_3M, PTS_12M, RB_3M, RB_12M). Others indicate the sum of the relative percentages of genera that represented less than 0.5% of the total reads attributed to the phylum. Unidentified indicates the relative percentage of genera which could not be identified to the corresponding taxonomic level.

| **Actinobacteria_Genus** | **PTS_3M**  **(%)** | **PTS_12M**  **(%)** | **RB_3M**  **(%)** | **RB_12M**  **(%)** |
| --- | --- | --- | --- | --- |
| Unidentified | 8.87 | 45.11 | 5.93 | 43.52 |
| *Clavibacter* | 0.66 | 0.02 | 3.32 | 0.02 |
| *Corynebacterium_1* | 4.36 | 0.03 | 1.65 | 0.03 |
| *Cryobacterium* | 1.10 | 0.00 | 2.34 | 0.06 |
| *Curtobacterium* | 7.45 | 0.00 | 1.34 | 0.01 |
| *Dermacoccus* | 4.82 | 0.02 | 0.68 | 0.00 |
| *Illumatobacter* | 6.23 | 52.02 | 1.27 | 52.30 |
| *Marisediminicola* | 0.04 | 0.00 | 3.45 | 0.07 |
| *Microbacterium* | 41.63 | 0.42 | 28.34 | 1.42 |
| *Nocardioides* | 0.79 | 0.00 | 1.11 | 0.01 |
| *Propionibacterium* | 8.03 | 0.84 | 44.94 | 0.31 |
| *Turicella* | 0.48 | 0.04 | 1.16 | 0.00 |
| Others | 15.54 | 1.50 | 4.47 | 2.27 |

**Figures S6A, S6B**


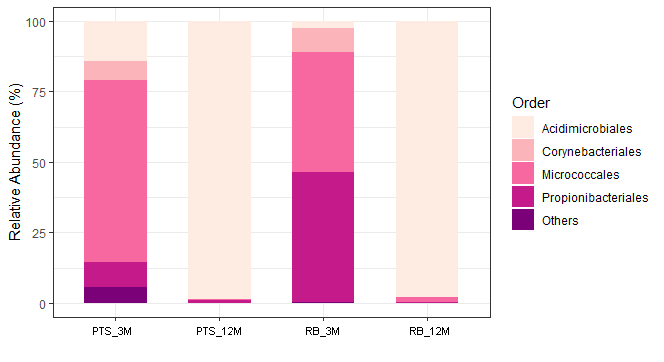

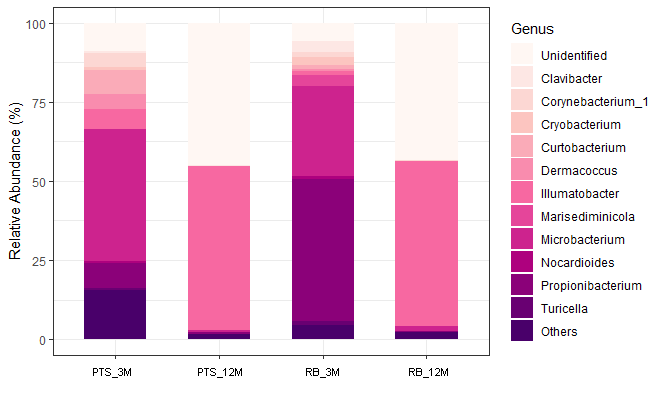


**A**

**B**

**Figure S6.** Relative abundance (%) at order **(A)** and genus **(B)** level of Actinobacteria in the Antarctic marine microbial biofilm communities (PTS_3M, PTS_12M, RB_3M, RB_12M). Others indicate the sum of the relative percentages of orders or genera that represented less than 0.5% of the total reads attributed to the phylum. Unidentified indicates the relative percentage of orders or genera which could not be identified to the corresponding taxonomic level.

**Table S8A, S8B, S8C**

**Table S8A.** Relative abundance (%) at class level of Firmicutes in the Antarctic marine microbial biofilm communities (PTS_3M, PTS_12M, RB_3M, RB_12M). Others indicate the sum of the relative percentages of classes that represented less than 0.5% of the total reads attributed to the phylum.

| **Firmicutes_Class** | **PTS_3M**  **(%)** | **PTS_12M**  **(%)** | **RB_3M**  **(%)** | **RB_12M**  **(%)** |
| --- | --- | --- | --- | --- |
| *Bacilli* | 93.38 | 55.39 | 81.49 | 74.07 |
| *Clostridia* | 0.27 | 36.92 | 14.32 | 16.66 |
| *Erysipelotrichia* | 6.35 | 7.70 | 4.19 | 9.26 |
| Others | 0.00 | 0.00 | 0.00 | 0.00 |

**Table S8B.** Relative abundance (%) at order level of Firmicutes in the Antarctic marine microbial biofilm communities (PTS_3M, PTS_12M, RB_3M, RB_12M). Others indicate the sum of the relative percentages of orders that represented less than 0.5% of the total reads attributed to the phylum.

| **Firmicutes_Order** | **PTS_3M**  **(%)** | **PTS_12M**  **(%)** | **RB_3M**  **(%)** | **RB_12M**  **(%)** |
| --- | --- | --- | --- | --- |
| *Bacillales* | 74.33 | 41.54 | 72.04 | 61.12 |
| *Clostridiales* | 0.27 | 32.31 | 12.98 | 16.66 |
| *Erysipelotrichales* | 6.35 | 7.70 | 4.19 | 9.26 |
| *Halanaerobiales* | 0.00 | 4.62 | 1.33 | 0.00 |
| *Lactobacillales* | 19.05 | 13.85 | 9.45 | 12.96 |
| Others | 0.00 | 0.00 | 0.00 | 0.00 |

**Table S8C.** Relative abundance (%) at genus level of Firmicutes in the Antarctic marine microbial biofilm communities (PTS_3M, PTS_12M, RB_3M, RB_12M). Others indicate the sum of the relative percentages of genera that represented less than 0.5% of the total reads attributed to the phylum.

| **Firmicutes_Genus** | **PTS_3M**  **(%)** | **PTS_12M**  **(%)** | **RB_3M**  **(%)** | **RB_12M**  **(%)** |
| --- | --- | --- | --- | --- |
| *Alloiococcus* | 0.00 | 4.62 | 0.98 | 0.00 |
| *Anaerobacillus* | 1.61 | 1.54 | 12.31 | 1.85 |
| *Anaerococcus* | 0.27 | 0.00 | 0.65 | 0.00 |
| *Bacillus* | 7.15 | 3.08 | 20.11 | 12.96 |
| *Blautia* | 0.00 | 3.08 | 3.23 | 3.70 |
| *Caminicella* | 0.00 | 12.31 | 2.35 | 0.00 |
| *Clostridium_sensu_stricto_13* | 0.00 | 0.00 | 4.19 | 0.00 |
| *Desulfitibacter* | 0.00 | 9.23 | 1.24 | 5.55 |
| *Erysipelothrix* | 6.35 | 7.70 | 4.19 | 9.26 |
| *Faecalibacterium* | 0.00 | 7.70 | 1.26 | 7.41 |
| *Gemella* | 2.51 | 1.54 | 0.61 | 0.00 |
| *Halanaerobium* | 0.00 | 4.62 | 1.33 | 0.00 |
| *Staphylococcus* | 63.06 | 35.39 | 38.92 | 46.31 |
| *Streptococcus* | 11.99 | 7.70 | 8.47 | 9.25 |
| Others | 7.07 | 1.54 | 0.17 | 3.70 |

**Figures S7A, S7B**


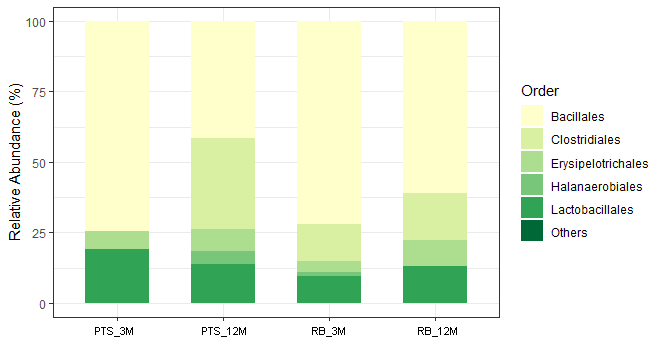

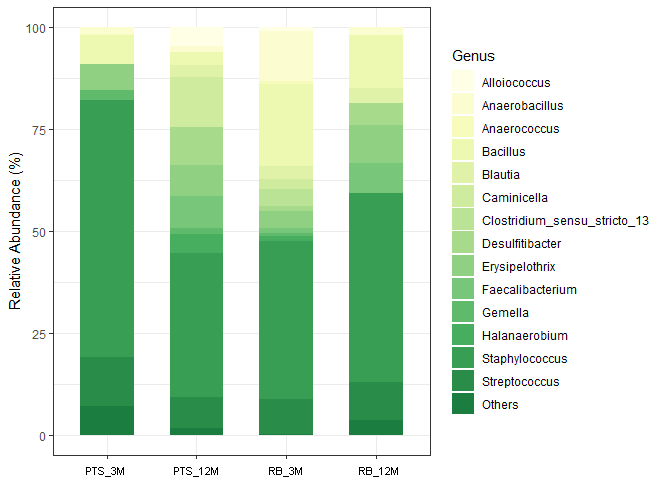


**A**

**B**

**Figure S7.** Relative abundance (%) at order **(A)** and genus **(B)** level of Firmicutes in the Antarctic marine microbial biofilm communities (PTS_3M, PTS_12M, RB_3M, RB_12M). Others indicate the sum of the relative percentages of orders or genera that represented less than 0.5% of the total reads attributed to the phylum.

**Table S9A, S9B**

**Table S9A.** Relative (%) abundance of potentially pathogenic bacterial genera in the Antarctic marine microbial biofilm communities (PTS_3M, PTS_12M, RB_3M, RB_12M). Others indicate the sum of the relative percentages of genera which are not included in the potential pathogen list.

|  | **PTS_3M**  **(%)** | **PTS_12M**  **(%)** | **RB_3M**  **(%)** | **RB_12M**  **(%)** |
| --- | --- | --- | --- | --- |
| Pathogenic genera | 4.11 | 0.33 | 27.49 | 0.14 |
| Others | 95.89 | 99.67 | 72.51 | 99.86 |

**Table S9B.** Absolute (Reads) abundance of potentially pathogenic bacterial genera in the Antarctic marine microbial biofilm communities (PTS_3M, PTS_12M, RB_3M, RB_12M). Others indicate the sum of the reads of potential pathogenic genera that represented less than 0.5% of the total reads.

| **Pathogenic_genera** | **PTS_3M**  **(Reads)** | **PTS_12M**  **(Reads)** | **RB_3M**  **(Reads)** | **RB_12M**  **(Reads)** |
| --- | --- | --- | --- | --- |
| *Achromobacter* | 39 | 1 | 604 | 0 |
| *Acinetobacter* | 210 | 12 | 2818 | 18 |
| *Bacillus* | 80 | 2 | 3368 | 7 |
| *Escherichia-Shigella* | 84 | 3 | 854 | 8 |
| *Propionibacterium* | 367 | 117 | 33260 | 33 |
| *Providencia* | 0 | 0 | 719 | 0 |
| *Pseudomonas* | 1061 | 71 | 7719 | 29 |
| *Sphingomonas* | 1631 | 363 | 1008 | 90 |
| *Staphylococcus* | 705 | 23 | 6516 | 25 |
| *Streptococcus* | 134 | 5 | 1419 | 5 |
| Others | 104 | 7 | 781 | 17 |

**Figure S8**





**Figure S8.** Absolute (Reads) abundance of potentially pathogenic genera in the Antarctic marine microbial biofilm communities (PTS_3M, PTS_12M, RB_3M, RB_12M). Others indicate the sum of the reads of potential pathogenic genera that represented less than 0.5% of the total reads.

**Table S10**

**Table S10.** Bacterial strains isolated from Antarctic marine biofilm samples collected in Terra Nova Bay (Ross Sea, Antarctica) on polyvinyl chloride panels at -5 m (86). Strain identification number, sampling site and colonization time (3M – 3 months, January, 12M – 12 months, November), culture medium (Marine Agar, MA; Isolation Streptomyces Project, ISP; Chitin Agar, CHA, Antarctic Bacterium Medium, ABM; Oatmeal Agar, OAT; * indicates addition of 20 g/L artificial sea salt from Haquoss, Aquarialand, Turin, Italy), sample treatment (+ indicates heat treatment at 55 °C for 5 min, - indicates no heat treatment), sample dilution and incubation temperatures of the plates during the isolation procedure are reported. Details about the colour of the colonies, their microscopical morphological classification (unicellular, Unicel; filamentous Filam), Gram staining (+ indicates Gram-positive, - indicates Gram-negative) and halophilicity (H indicates halophile, T indicates halotolerant) are also reported.

| **Strain ID Number** | **Site** | **Colonization Time** | **Isolation Medium** | **Sample Treatment** | **Sample Dilution** | **Incubation Temperature**  **(°C)** | **Colony Colour** | **Morpho**  **logical**  **Classification** | **Gram Staining** | **Halo**  **philicity** |
| --- | --- | --- | --- | --- | --- | --- | --- | --- | --- | --- |
| **B2S** | RB | 3M | MA | - | - | 28 | Yellow | Unicel | + | T |
| **B3S** | RB | 3M | ISP4* | - | - | 28 | White | Unicel | + | T |
| **B4S** | RB | 3M | ISP4* | - | - | 28 | Orange | Unicel | + | T |
| **B6S** | RB | 3M | MA | + | - | 28 | White | Unicel | + | H |
| **B8S** | RB | 3M | ISP2* | + | - | 28 | White | Unicel | + | T |
| **B9S** | RB | 3M | ISP4* | + | - | 28 | Orange | Unicel | + | H |
| **B10S** | PTS | 3M | MA | + | - | 28 | White-grey | Unicel | + | H |
| **B12S** | PTS | 3M | ISP2* | - | - | 28 | White | Unicel | + | H |
| **B13S** | PTS | 3M | ISP6* | - | - | 28 | Grey | Unicel | + | T |
| **B15S** | PTS | 3M | ISP4* | + | - | 28 | Yellow | Unicel | + | T |
| **B17S** | PTS | 3M | ISP5* | - | 10^-3^ | 28 | Yellow | Unicel | + | T |
| **B18S** | PTS | 3M | ISP6* | + | 10^-3^ | 28 | Dark grey | Filam | + | T |
| **B24S** | RB | 3M | ISP5* | + | 10^-3^ | 28 | White | Unicel | + | T |
| **B28S** | RB | 3M | ISP4* | - | - | 20 | White-grey | Filam | + | T |
| **B29S** | RB | 3M | ISP4* | - | - | 20 | White | Filam | + | T |
| **B31S** | RB | 3M | ISP4* | - | - | 20 | Brown | Filam | + | T |
| **B32S** | RB | 3M | ISP4* | - | - | 20 | Dark grey | Filam | + | T |
| **B33S** | RB | 3M | ISP4* | - | - | 20 | White | Filam | + | T |
| **C33** | RB | 3M | ISP4* | - | - | 20 | Yellow | Unicel | + | T |
| **B34S** | RB | 3M | ISP4* | - | - | 20 | Dark grey | Filam | + | T |
| **B36S** | RB | 3M | ISP4* | - | - | 20 | Brown | Unicel | + | H |
| **B37S** | RB | 3M | ISP4* | - | - | 20 | Brown | Unicel | + | H |
| **B38S** | RB | 3M | ISP4* | - | - | 20 | Brown | Filam | + | T |
| **B39S** | PTS | 3M | ISP6* | - | - | 20 | White | Filam | + | T |
| **B40S** | PTS | 3M | MA | + | 10^-3^ | 20 | Brown | Filam | + | T |
| **B42S** | RB | 3M | ISP5* | - | - | 20 | White | Filam | + | T |
| **B43S** | RB | 3M | ISP6* | - | - | 20 | Orange | Unicel | + | T |
| **B44S** | RB | 3M | ISP5* | + | - | 20 | White | Filam | + | T |
| **B46S** | PTS | 3M | ISP4* | - | - | 20 | White-grey | Filam | + | T |
| **B47S** | PTS | 3M | ISP4* | - | - | 20 | White | Unicel | + | T |
| **B48S** | PTS | 3M | ISP4* | - | - | 20 | White | Filam | + | T |
| **B50S** | PTS | 3M | ISP4* | - | - | 20 | Brown | Unicel | + | H |
| **B51S** | PTS | 3M | ISP4* | - | - | 20 | White | Unicel | + | T |
| **B52S** | PTS | 3M | ISP4* | - | - | 20 | White | Filam | + | T |
| **B54S** | PTS | 3M | ISP4* | - | - | 20 | Pink | Unicel | + | T |
| **B55S** | PTS | 3M | ISP4* | - | - | 20 | White | Filam | + | T |
| **B59S** | RB | 3M | ISP6* | + | 10^-3^ | 20 | White | Unicel | + | T |
| **B60S** | PTS | 3M | ISP5* | - | 10^-6^ | 20 | White | Filam | + | T |
| **B62S** | PTS | 3M | ISP4* | + | 10^-6^ | 20 | Grey | Filam | + | T |
| **B64S** | RB | 3M | ISP4* | + | 10^-6^ | 20 | Grey | Unicel | + | H |
| **B65S** | PTS | 3M | MA | + | - | 20 | White | Unicel | + | H |
| **B66S** | PTS | 3M | ISP5* | + | - | 20 | Yellow | Unicel | + | T |
| **B67S** | RB | 3M | MA | - | - | 20 | White | Unicel | + | T |
| **B69S** | PTS | 3M | ISP6* | + | 10^-3^ | 20 | White | Unicel | + | T |
| **B70S** | RB | 3M | ISP2* | - | 10^-3^ | 20 | Yellow | Unicel | + | T |
| **B71S** | RB | 3M | ISP2* | + | 10^-3^ | 20 | White | Unicel | + | T |
| **B74S** | PTS | 3M | MA | - | 10^-6^ | 20 | Orange | Unicel | - | H |
| **B75S** | RB | 3M | ISP2* | + | - | 28 | Grey | Unicel | + | H |
| **B76S** | RB | 3M | ISP6* | + | 10^-3^ | 20 | Orange | Unicel | + | H |
| **B78S** | RB | 3M | ISP7* | - | - | 20 | White | Unicel | + | T |
| **B79S** | RB | 3M | ISP5* | - | 10^-3^ | 20 | Orange | Unicel | + | H |
| **B80S** | RB | 3M | ISP6* | - | - | 4 | Brown | Unicel | + | H |
| **B81S** | RB | 3M | ISP4* | - | - | 4 | Orange | Unicel | + | T |
| **B82S** | RB | 3M | ISP4* | - | - | 4 | Orange | Unicel | + | T |
| **B83S** | RB | 3M | ISP4* | - | - | 4 | White | Unicel | + | T |
| **B84S** | RB | 3M | ISP7* | - | - | 4 | Orange | Unicel | + | H |
| **B85S** | RB | 3M | ISP7* | - | - | 4 | Orange | Unicel | + | T |
| **B86S** | RB | 3M | ISP7* | - | - | 4 | Orange | Unicel | + | T |
| **B87S** | RB | 3M | ISP5* | - | - | 4 | Orange | Unicel | + | H |
| **B1** | RB | 3M | ISP4 | - | - | 28 | Orange | Unicel | + | T |
| **B2** | RB | 3M | ISP4 | - | - | 28 | Grey | Unicel | + | T |
| **B11** | RB | 3M | CHA | - | 10^-6^ | 28 | Orange | Unicel | + | T |
| **B16** | RB | 3M | ABM | + | - | 20 | White | Unicel | + | T |
| **B17** | RB | 3M | ISP6 | - | - | 20 | Yellow | Unicel | - | T |
| **B18** | RB | 3M | ISP6 | - | - | 20 | Yellow | Unicel | - | T |
| **B20** | RB | 3M | ISP4 | - | - | 20 | Yellow | Unicel | + | T |
| **B21** | RB | 3M | ISP5 | + | - | 20 | White | Unicel | + | T |
| **B22** | RB | 3M | ISP5 | + | - | 20 | White | Unicel | + | T |
| **B32** | RB | 3M | CHA | - | 10^-6^ | 20 | White | Unicel | + | T |
| **B34** | PTS | 3M | ISP2 | + | 10^-9^ | 20 | Yellow | Unicel | - | T |
| **B36** | PTS | 3M | ISP6 | - | 10^-9^ | 20 | White | Unicel | - | T |
| **B44** | RB | 3M | ABM | - | 10^-6^ | 4 | White | Unicel | + | T |
| **B45** | RB | 3M | ISP6 | - | 10^-6^ | 4 | White | Unicel | + | T |
| **B46** | PTS | 3M | ISP7 | + | 10^-9^ | 4 | White | Unicel | + | T |
| **B47** | RB | 3M | ISP5 | - | - | 4 | White | Unicel | + | T |
| **B235S** | PTS | 12M | ISP4* | - | - | 20 | Orange | Unicel | + | T |
| **B236S** | PTS | 12M | MA | - | - | 20 | Orange | Unicel | - | T |
| **B247S** | RB | 12M | ISP4* | - | - | 20 | Yellow | Unicel | - | T |
| **B248S** | RB | 12M | ISP4* | - | - | 20 | White | Unicel | + | T |
| **B249S** | RB | 12M | ISP4* | - | - | 20 | Beige | Unicel | + | T |
| **B250S** | RB | 12M | ISP4* | - | - | 20 | Grey | Unicel | - | T |
| **B251S** | RB | 12M | ISP4* | - | - | 20 | Yellow | Unicel | + | T |
| **B252S** | RB | 12M | MA | - | - | 20 | Orange | Unicel | + | T |
| **B253S** | RB | 12M | ISP4* | - | - | 20 | White | Unicel | - | H |
| **B254S** | RB | 12M | ISP4* | - | - | 20 | Beige | Unicel | + | T |
| **B255S** | RB | 12M | MA | - | - | 20 | Beige | Filam | + | T |

**Table S11**

**Table S11.** Hydrolytic and oxidative enzyme activities detected amongst the Antarctic marine biofilm bacterial isolates (86) through degradation and/or colorimetric enzyme assays on agar plates. The strains are classified at the level of phylum and genus. + indicates that the corresponding enzyme activity was detectable, while – indicates that it was not detectable.

| **Phylum** | **Genus** | **Strain ID Number** | **Proteolytic** | **Lipolytic** | **Amylolytic** | **Chitinolytic** | **Cellulolytic** | **ABTS** | **Azure B** |
| --- | --- | --- | --- | --- | --- | --- | --- | --- | --- |
| *Firmicutes* | *Bacillus* | B3S | **-** | **+** | **-** | **-** | **-** | **-** | **-** |
|  |  | B8S | **+** | **-** | **+** | **+** | **-** | **-** | **-** |
|  |  | B46 | **+** | **-** | **+** | **+** | **-** | **-** | **+** |
|  |  | B36S | **-** | **-** | **-** | **-** | **-** | **-** | **-** |
|  |  | B37S | **-** | **-** | **-** | **-** | **-** | **-** | **-** |
|  | *Peribacillus* | B47S | **+** | **-** | **+** | **-** | **-** | **-** | **-** |
|  |  | B54S | **+** | **-** | **+** | **-** | **-** | **-** | **-** |
|  |  | B70S | **+** | **+** | **+** | **-** | **-** | **-** | **-** |
|  | *Lysinibacillus* | B254S | **+** | **-** | **+** | **-** | **-** | **-** | **-** |
|  | *Oceanobacillus* | B10S | **+** | **+** | **-** | **-** | **-** | **+** | **-** |
|  |  | B50S | **+** | **-** | **-** | **-** | **-** | **-** | **-** |
|  |  | B64S | **+** | **-** | **-** | **-** | **-** | **-** | **-** |
|  | *Planomicrobium* | B4S | **+** | **+** | **-** | **-** | **-** | **-** | **-** |
|  |  | B59S | **+** | **+** | **-** | **-** | **-** | **-** | **-** |
|  | *Planococcus* | B84S | **+** | **-** | **-** | **-** | **-** | **-** | **-** |
|  |  | B87S | **+** | **-** | **-** | **-** | **-** | **-** | **-** |
|  | *Sporosarcina* | B6S | **-** | **-** | **-** | **-** | **-** | **-** | **-** |
|  |  | B12S | **-** | **-** | **-** | **-** | **-** | **-** | **-** |
|  |  | B67S | **+** | **-** | **-** | **-** | **-** | **-** | **-** |
|  |  | B85S | **+** | **-** | **-** | **-** | **-** | **-** | **-** |
|  |  | B86S | **+** | **-** | **-** | **-** | **-** | **-** | **-** |
|  |  | B1 | **-** | **-** | **-** | **-** | **-** | **-** | **-** |
|  |  | B45 | **-** | **-** | **-** | **-** | **-** | **-** | **-** |
|  | *Paenisporosarcina* | B65S | **+** | **-** | **-** | **-** | **-** | **-** | **-** |
|  |  | B80S | **+** | **-** | **-** | **-** | **-** | **-** | **-** |
|  |  | B82S | **+** | **+** | **-** | **-** | **-** | **-** | **-** |
|  |  | B2 | **+** | **+** | **-** | **-** | **-** | **-** | **-** |
|  | *Staphylococcus* | B71S | **+** | **-** | **+** | **-** | **-** | **-** | **-** |
| *Actinobacteria* | *Rhodococcus* | B2S | **+** | **-** | **-** | **-** | **-** | **+** | **-** |
|  |  | B15S | **+** | **-** | **-** | **-** | **-** | **-** | **-** |
|  |  | B17S | **+** | **-** | **-** | **-** | **-** | **-** | **-** |
|  | *Arthrobacter* | B13S | **+** | **-** | **-** | **-** | **-** | **-** | **-** |
|  |  | B69S | **+** | **-** | **-** | **-** | **-** | **-** | **-** |
|  |  | B24S | **-** | **-** | **-** | **-** | **-** | **-** | **-** |
|  |  | B81S | **-** | **-** | **-** | **-** | **-** | **-** | **-** |
|  |  | B43S | **+** | **+** | **+** | **-** | **-** | **-** | **-** |
|  |  | B83S | **+** | **+** | **+** | **-** | **-** | **-** | **-** |
|  |  | B51S | **+** | **-** | **-** | **-** | **-** | **-** | **-** |
|  |  | B66S | **+** | **+** | **-** | **-** | **-** | **-** | **-** |
|  |  | B78S | **-** | **-** | **-** | **-** | **-** | **-** | **-** |
|  | *Pseudoarthrobacter* | B75S | **-** | **-** | **-** | **-** | **-** | **-** | **-** |
|  |  | B248S | **-** | **-** | **-** | **-** | **-** | **-** | **-** |
|  |  | B249S | **+** | **+** | **+** | **-** | **-** | **-** | **-** |
|  | *Plantibacter* | B11 | **-** | **-** | **-** | **-** | **-** | **-** | **-** |
|  |  | B47 | **-** | **-** | **-** | **-** | **-** | **-** | **-** |
|  | *Brachybacterium* | B16 | **-** | **-** | **-** | **-** | **-** | **-** | **-** |
|  |  | B21 | **-** | **-** | **-** | **-** | **-** | **-** | **-** |
|  |  | B22 | **-** | **-** | **-** | **-** | **-** | **-** | **-** |
|  |  | B32 | **-** | **-** | **-** | **-** | **-** | **-** | **-** |
|  | *Brevibacterium* | B44 | **+** | **-** | **-** | **-** | **-** | **-** | **-** |
|  |  | B235S | **+** | **-** | **-** | **-** | **-** | **-** | **-** |
|  | *Micrococcus* | B20 | **-** | **+** | **-** | **-** | **-** | **-** | **+** |
|  |  | B252S | **-** | **+** | **-** | **-** | **-** | **-** | **-** |
|  | *Dietzia* | B9S | **-** | **-** | **-** | **-** | **-** | **-** | **-** |
|  |  | B76S | **-** | **-** | **-** | **-** | **-** | **-** | **-** |
|  |  | B79S | **-** | **-** | **-** | **-** | **-** | **-** | **-** |
|  | *Streptomyces* | B18S | **+** | **-** | **+** | **-** | **-** | **-** | **-** |
|  |  | B28S | **-** | **-** | **+** | **+** | **-** | **-** | **+** |
|  |  | B29S | **+** | **-** | **-** | **+** | **-** | **-** | **+** |
|  |  | B31S | **+** | **-** | **-** | **+** | **-** | **-** | **-** |
|  |  | B32S | **+** | **-** | **-** | **+** | **-** | **-** | **-** |
|  |  | B33S | **+** | **-** | **-** | **+** | **-** | **-** | **-** |
|  |  | B34S | **+** | **-** | **+** | **-** | **-** | **-** | **-** |
|  |  | B255S | **-** | **+** | **+** | **+** | **-** | **+** | **-** |
|  |  | B38S | **+** | **-** | **-** | **+** | **-** | **-** | **-** |
|  |  | B39S | **+** | **-** | **-** | **-** | **-** | **-** | **-** |
|  |  | B40S | **+** | **-** | **-** | **-** | **-** | **-** | **-** |
|  |  | B42S | **+** | **-** | **-** | **+** | **-** | **-** | **-** |
|  |  | B44S | **-** | **+** | **-** | **-** | **-** | **-** | **-** |
|  |  | B46S | **+** | **-** | **+** | **+** | **-** | **-** | **-** |
|  |  | B48S | **+** | **+** | **-** | **-** | **-** | **-** | **-** |
|  |  | B52S | **+** | **-** | **-** | **-** | **-** | **-** | **-** |
|  |  | B55S | **+** | **-** | **-** | **-** | **-** | **-** | **-** |
|  |  | B60S | **+** | **-** | **-** | **+** | **-** | **-** | **-** |
|  |  | B62S | **+** | **+** | **+** | **-** | **-** | **-** | **-** |
| *Gamma-*  *Proteobacteria* | *Acinetobacter* | C33 | **-** | **-** | **-** | **+** | **-** | **-** | **-** |
|  | *Psychrobacter* | B74S | **-** | **-** | **-** | **-** | **-** | **-** | **-** |
|  |  | B34 | **-** | **-** | **-** | **-** | **-** | **-** | **-** |
|  |  | B36 | **-** | **-** | **-** | **-** | **-** | **-** | **-** |
|  |  | B236S | **-** | **-** | **+** | **-** | **-** | **-** | **-** |
|  |  | B247S | **-** | **-** | **-** | **-** | **-** | **-** | **-** |
|  |  | B17 | **-** | **-** | **-** | **-** | **-** | **-** | **-** |
|  |  | B18 | **-** | **-** | **-** | **-** | **-** | **-** | **-** |
|  |  | B253S | **-** | **+** | **+** | **-** | **-** | **-** | **-** |
| *Alpha-*  *Proteobacteria* | *Paracoccus* | B250S | **-** | **-** | **-** | **-** | **-** | **-** | **-** |
|  |  | B251S | **-** | **+** | **+** | **-** | **-** | **-** | **-** |

**Table S12**

**Table S12.** List of bacterial genera identified through either the culture-independent approach (16S rRNA gene amplicon sequencing, 356), the culture-dependent one (16S rRNA gene amplification and sequencing from genomic DNA on isolated strains, 11), or by both of them (shared, 10).

| **Unique Metagenomic Genera** |
| --- |
| **A**  Achromobacter, Acidibacter, Acidiphilium, Acidovorax, Actibacter, Actinomyces, Actinoplanes, Aeromonas, Aestuariihabitans, Afipia, Ahrensia, Albidiferax, Algibacter, Algimonas, Alloiococcus, Alpinimonas, Altererythrobacter, Alteromonas, Aminobacter, Amylibacter, Anaerobacillus, Anaerobaculum, Anaerococcus, Antarctobacter, Aquamicrobium, Aquibacter, Arcobacter, Arenibacter, Arenicella, Arenimonas, Ascidiaceihabitans, Aureimonas, Aureispira |
| **B**  Bdellovibrio, Bergeyella, Bizionia, Blastomonas, Blastopirellula, Blautia, Bordetella, Boseongicola, Bradyrhizobium, Brevundimonas, Byssovorax, Bythopirellula |
| **C**  Caldithrix, Caminicella, Campylobacter, Candidatus_Branchiomonas, Candidatus_Koribacter, Candidatus_Microthrix, Candidatus_Thiobios, Caulobacter, Cedecea, Cellulomonas, Cellvibrio, Chlorochromatium, Chryseobacterium, Chthoniobacter, Clavibacter, Clostridium, Cocleimonas, Colwellia, Corynebacterium Crenotalea, Croceicoccus, Crocinitomix, Cryobacterium, Cryomorpha, Curtobacterium, Curvibacter, Cycloclasticus |
| **D**  Dasania, Deefgea, Defluviimonas, Deinococcus, Delftia, Dermacoccus, Desulfitibacter, Desulfocapsa, Desulforhopalus, Devosia, Dinoroseobacter, Diplosphaera, Dokdonia, Dolosigranulum, Dongia, Duganella |
| **E**  Ectothiorhodospira, Ekhidna, Empedobacter, Enhydrobacter, Ensifer, Enterobacter, Enterococcus, Erwinia, Erysipelothrix, Erythrobacter, Escherichia-Shigella, Euzebyella |
| **F**  Fabibacter, Faecalibacterium, Ferritrophicum, Ferruginibacter, Filomicrobium, Flaviramulus, Flavobacterium, Flexithrix, Fluviicola, Formosa, Francisella, Fretibacter, Frigoribacterium, Fulvivirga |
| **G**  Gaetbulibacter, Gemella, Gemmobacter, Geojedonia, Glaciecola, Gordonia, Granulosicoccus |
| **H**  Haemophilus, Halanaerobium, Haliangium, Haliea, Halioglobus, Halobacteriovorax, Halocynthiibacter, Haloferula, Halomonas, Hellea, Hoeflea, Hydrogenophaga,Hydrotalea, Hymenobacter, Hyphomicrobium |
| **I**  Illumatobacter, Iodobacter |
| **J**  Janibacter, Jannaschia, Janthinobacterium, |
| **K**  Kallotenue, Kangiella, Kiloniella, Klebsiella, Kluyvera, Kocuria, Kordia, |
| **L**  Labedella, Lachnoclostridium, Lacinutrix, Lautropia, Leifsonia, Lentibacter, Lentisphaera, Leptothrix, Leptotrichia, Leucobacter, Leucothrix, Lewinella, Limnobacter, Limnohabitans, Litoreibacter, Litorimicrobium, Litorimonas, Loktanella, Luminiphilus, Luteolibacter, Lutibacter, Lutimonas, Lysinimonas |
| **M**  Magnetospira, Malikia, Maribacter, Marinicella, Marinifilum, Marinomonas, Marinosulfonomonas, Marinovum, Marisediminicola, Maritimimonas, Marixanthomonas, Massilia, Meganema, Mesoflavibacter, Mesorhizobium, Methanobacterium, Methanoculleus, Methanothermobacter, Methylobacterium, Methyloferula, Methylomonas, Methylorosula, Methylotenera, Microbacterium, Microcoleus, Mitsuaria, Moraxella, Morganella, Moritella, Mucilaginibacter, Mycobacterium |
| **N**  Nakamurella, Nautella, Neisseria, Neptunomonas, Nereida, Nitratireductor, Nitrosococcus, Nitrosomonas, Nitrospira, Nocardioides, Novosphingobium |
| **O**  Oceanicoccus, Oceanicola, Oceaniovalibus, Oceaniserpentilla, Oceanobacterium, Octadecabacter, Oleiphilus, Oleispira, Olleya, Opitutus, Ornithinibacillus, Ottowia, Owenweeksia |
| **P**  Palleronia, Paraglaciecola, Parapedobacter, Paucibacter, Pedobacter, Pedomicrobium, Pelagicola, Pelagimonas, Pelomonas, Peredibacter, Persicirhabdus, Perspicuibacter, Petrotoga, Phaeobacter, Phaeodactylibacter, Phenylobacterium, Photobacterium, Phycicola, Phyllobacterium, Pibocella, Pirellula, Planctomyces, Planktomarina, Planktotalea, Planomicrobium, Polaribacter, Polaromonas, Polymorphobacter, Polynucleobacter, Portibacter, Pricia, Primorskyibacter, Prochlorococcus, Profundibacterium, Propionibacterium, Providencia, Pseudahrensia, Pseudofulvibacter, Pseudohaliea, Pseudomonas, Pseudophaeobacter, Pseudoruegeria, Pseudospirillum, Pseudoxanthomonas, Psychrilyobacter, Psychromonas, Psychroserpens, Puniceibacterium |
| **R**  Ramlibacter, Reichenbachiella, Reyranella, Rhizobacter, Rhizobium, Rhizomicrobium, Rhodanobacter, Rhodobacter, Rhodoferax, Rhodopirellula, Robiginitomaculum, Roseateles, Roseibacillus, Roseibacterium, Roseivivax, Roseobacter, Roseomonas, Roseovarius, Rothia, Rubidimonas, Rubricoccus, Rubripirellula, Rubritalea, Rubrivirga, Ruegeria, Rugamonas |
| **S**  Sabulilitoribacter, Sagittula, Salinibacterium, Salinihabitans, Salinirepens, Schleiferia, Sedimentitalea, Sediminibacterium, Seohaeicola, Serratia, Shewanella, Shinella, Simiduia, Simplicispira, Sneathiella, Solitalea, Sphingobium, Sphingomonas, Sphingopyxis, Sphingorhabdus, Spirochaeta, Spirosoma, Spongiibacter, Stenotrophomonas, Streptococcus, Subsaxibacter, Sufflavibacter, Sulfitobacter, Sulfurimonas, Sulfurospirillum, Sulfurovum |
| **T**  Taibaiella, Tateyamaria, Tenacibaculum, Terrimonas, Thalassobacter, Thalassobius, Thermomonas, Thermovirga, Thioalkalivibrio, Thiomicrospira, Thiorhodospira, Tropicimonas, Turicella |
| **U**  Uliginosibacterium, Ulvibacter, Undibacterium |
| **V**  Vadicella, Variovorax |
| **W**  Wenyingzhuangia, Willamsia, Winogradskyella |
| **X**  Xanthomonas |
| **Y**  Yeosuana, Yersinia |
| **Z**  Zobellia |
| **Unique Isolation Genera** |
| **B**  Brachybacterium |
| **D**  Dietzia |
| **L**  Lysinibacillus |
| **O**  Oceanobacillus |
| **P**  Paenisporosarcina, Peribacillus, Planomicrobium, Plantibacter, Pseudoarthrobacter |
| **S**  Sporosarcina, Streptomyces |
| **Shared Genera** |
| **A**  Acinetobacter, Arthrobacter |
| **B**  Bacillus, Brevibacterium |
| **M**  Micrococcus |
| **P**  Paracoccus, Planococcus, Psychrobacter |
| **R**  Rhodococcus |
| **S**  Staphylococcus |

**Appendix References**

[1] Reddy, G. S., Aggarwal, R. K., Matsumoto, G. I., Shivaji, S. (**2000**). Arthrobacter flavus sp. nov., a psychrophilic bacterium isolated from a pond in McMurdo Dry Valley, Antarctica. International journal of systematic and evolutionary microbiology, **50**(4), 1553-1561. doi: 10.1099/00207713-50-4-1553

[2] Hsu, S. C., Lockwood, J. L. (**1975**). Powdered chitin agar as a selective medium for enumeration of actinomycetes in water and soil. *Applied microbiology*, **29**(3), 422-426. doi: 10.1128/am.29.3.422-426.1975

[3] Marcone, G. L., Binda, E., Reguzzoni, M., Gastaldo, L., Dalmastri, C., Marinelli, F. (**2017**). Classification of *Actinoplanes* sp. ATCC 33076, an actinomycete that produces the glycolipodepsipeptide antibiotic ramoplanin, as *Actinoplanes ramoplaninifer* sp. nov. International journal of systematic and evolutionary microbiology, **67**(10), 4181-4188. doi: 10.1099/ijsem.0.002281

[4] Caruso, G., Dell’Acqua, O., Caruso, R., Azzaro, M. (**2022**). Phenotypic characterization of bacterial isolates from marine waters and plastisphere communities of the Ross Sea (Antarctica). *Journal of clinical microbiology and biochemical technology,* **8**(1), 1-9. doi: 10.17352/jcmbt.000048

[5] Baxter M., Sieburth J. M. (**1984**). Metabolic and ultrastructural response to glucose of two eurytrophic bacteria isolated from seawater at different enriching concentrations. *Applied environmental microbiology,* **47**(1), 31-38. doi: 10.1128/aem.47.1.31-38.1984
